# Supplementary material for: Qualitative exploration of the constraints on mothers’ and pregnant women’s ability to turn available services into nutrition benefits in a low-resource urban setting, South Africa
Source: BMJ Open. 2023 Nov 22;13(11):e073716. doi: 10.1136/bmjopen-2023-073716 (PMC10668265; doi:10.1136/bmjopen-2023-073716)
Supplement: Supplementary data [file bmjopen-2023-073716supp001.pdf]

Supplemental file 1 - Total number of focus groups held by sociodemographic characteristics of participants

FGD no.	Participants (n)	Age (years) (min-max)	Pregnancy status	Participants with a household income <=3000 ZAR/month (n)	Participants with >=2 children (n)	Mean number of household members (SD)	Participants (n) per selected community health centres
1	7	22-40	Pregnant	5	1	4 (2.7)	Chiawelo (1), Itireleng (1), Lillian Ngoyi (-), Mofolo (3), Zola (2)
2	8	22-40	Not pregnant	4	7	5 (2.4)	Chiawelo (3), Itireleng (3), Lillian Ngoyi (-), Mofolo (2), Zola (2)
3	7	19-47	Not pregnant	6	5	4 (1.6)	Chiawelo (1), Itireleng (1), Lillian Ngoyi (1), Mofolo (3), Zola (1)
4	8	18-37	Pregnant	7	3	5 (2)	Chiawelo (1), Itireleng (3), Lillian Ngoyi (2), Mofolo (1), Zola (1)
5	8	26-49	Not pregnant	5	4	6 (2.7)	Chiawelo (1), Itireleng (2), Lillian Ngoyi (2), Mofolo (1), Zola (2)
6	7	22-48	Not pregnant	5	6	5 (2.8)	Chiawelo (1), Itireleng (3), Lillian Ngoyi (-), Mofolo (1), Zola (2)
7	6	19-42	Pregnant	3	2	4 (1.6)	Chiawelo (1), Itireleng (1), Lillian Ngoyi (2), Mofolo (2), Zola (-)

Erzse A, *et al. BMJ Open* 2023; 13:e073716. doi: 10.1136/bmjopen-2023-073716

Supplemental file 2 - Semi-structured interview guide for employees from community-based organizations

Guidance for interviewer:

- Thank the participant's willingness to participate.
- Introduce yourself and the project, including i) the purpose for the interview, ii) who is involved in the process, iii) why the i participant's cooperation is important in collecting the information, iv) what will happen with the collected information and how the community will benefit.
- Explain that participation is completely voluntary, and the participant is free to decline/withdraw before or even after starting the interview process. If the interviewee says no, this will not have any negative consequences for the participant.
- Ensure that participant has provided written consent to participate and audio record the interview.

Interview questions:

- Could you please introduce yourself, tell me a little bit about who you are, where you are from? (*Probe: age, education background, how long have they been working at the organization, any children, where did they grow up, where do they live now.)*
- Can you describe your role at the organization?
- Could you please tell me about the projects / services that the organization runs? (*Probe: How do these interventions work? Think of impact pathways and mechanism).*
- How does your organization decide which activities it runs?
- How do you measure the success of programmes that are delivered in this community? *(Probe: what are some of the indicators used).*
- Can you tell me about the community that your organization works with?
- What are the greatest challenges facing pregnant women or women with young children in the community you are working with?
  (Probes:
  - *What do you think some of the reasons are that the challenge, such as: _________ happen?*
  - *Can you think of someone who is struggling with _________(e.g., unemployment)? What stopped her from finding employment? What have they done to overcome this?)*
- You talked about challenges, such as: _________. How does this challenge affect nutrition for pregnant women and infants and young children in this community?
- One of the challenges you talked about that affect nutrition was: ________. What are some of the needs mothers have to overcome this challenge? Do you know of services and resources that can help? *(Probe if the focus is on government/medical solution*: *Is there anything that could help in the community?)*
- I would like to hear your opinion about the following scenario: Let's say that two mothers receive the same service *(repeat a service mentioned)*. One mom can fully benefit and have good nutrition. The other mom is not able to turn the service into nutrition benefit. Can you think of reasons why this could be?
  (*Probes:*
  - *What limits the mom's ability to ____?*
  - *Is this a common issue in your community?*
  - *How can moms overcome this challenge?)*
- Do any of these services exist or are there others in place?

(If participants find it difficult to answer, provide examples and probe: in some settings, mothers who e.g. receive home-energy assistance programme or housing stability programme improve their nutrition. What would be relevant in this community?)

- How could these interventions work? What are the ways that it could make a difference? *(Probe: impact pathways and mechanism)*
- In my study I am using the term "social need" is this a term that you are familiar with? And the community? What is your understanding of this term? What other terms are used that might mean the same thing?
- Do you have any additional comments or information you would like to share that I have not asked you?

Guidance for interviewer: thank the participant their time and contribution. Provide reimbursement and ensure receipt of fund sheet has been signed.

Supplemental file 3 - Focus group discussion guide

Blue words provide the focus group discussion facilitator directions. These are not meant to be read aloud. Black words are meant to be read aloud.

INTRODUCTION

As we begin, let me tell you a bit about todays' discussion. We will focus on mom's, including pregnant women, and child nutrition. More specifically, we will talk about issues that **help** or **limit** moms' ability to keep themselves and their children well-nourished in your community.

This discussion is the second step in a bigger research project. In the first step we asked community-based organizations in Soweto about their experience of the different challenges and needs moms have when it comes to nutrition. This is because poor nutrition is common in Soweto. Some moms and children lack of important vitamins, some are undernourished some have obesity.

Community-based organizations helped us understand that the government tries to provide services to make sure that moms and children can get good nutritional care and support. There is help at the clinic level through supplementation and nutrition education, and there is also help from the government through the child support grants. However, we learnt that not every mom can benefit from these services the same way, and not every mom and child will be well nourished. This is because we are not the same. We face different challenges and have different opportunities. And these affect our ability to buy and eat nutritious food.

Today, we want to understand these challenges and find solutions with you to improve the nutrition for moms and children.

There will be 2 parts to our discussion.

First, you will be asked to talk about challenges that affect the nutrition of moms and pregnant women living in this community. Second, you will be asked to think of some of the resources or services that exist in your area that might help meet these needs and concerns.

Erzse A, *et al. BMJ Open* 2023; 13:e073716. doi: 10.1136/bmjopen-2023-073716

There are no right or wrong answers. How does everyone feel about this so far? Do you have any questions? If not, please sign the consent form to participate and to audio record the discussion. Please write you name as well as your participant number on the forms.

Participants sing consent forms.

Before we begin, here are some ground rules. I am going to ask you keep your phone on silent/switch it off. I would also like to ask everyone, to speak as loud and clear as possible. Please, respect each other's opinions and give each other time to speak without disturbance. Only one person will be allowed to speak at a time. You can raise your hand if you have something to say and I will give you an opportunity. It is possible that you know someone who is here today. In this case, it is important that we all keep this discussion private.

Let participants know that you will start recording. Start recording. List place, date, time, group number and type (pregnant mothers or non-pregnant mothers).

PART 1

ICEBREAKER:
I'd like everyone to introduce themselves using your participant number and share how many kids you have, and what you heard about good nutrition from the clinic or anywhere else.

QUESTION 1:

As a next step, I would like to hear your opinion about the following scenario: Let's say that two mothers receive the same nutrition advice at the clinics. One mom is able to follow this advice and have good nutrition. The other mom is not able to do as she is told.

Can you think of reasons why this could be?

Probes: - What limits the mom's ability to follow the advice?
- *Is this a common issue in your community?*
- *How can moms overcome this challenge?*

QUESTION 2:

As we move on, I will read another scenario and ask you opinion.
It goes like: *"Your friend, Nomsa lives with her young child who has been losing weight. The supermarket to buy healthy food is an hour away by taxi. Nomsa is struggling to provide food for her hungry child. For months, they haven't eaten any fruits and vegetables, so they fill up with pap. This week she had to sell clothes to buy food."*

Is this something that happens in your community? Can you think of a solution? (if solution of "government" ask about local solutions).

Probes: - What do moms buy when they can't buy healthy food?
- *Can you think of someone who overcome this challenge?*

QUESTION 3:

We talked about X and Y that makes it difficult for a mother to have nutritious and healthy diet. What are other challenges can you think of that affect the nutrition of moms and pregnant women?

Erzse A, *et al. BMJ Open* 2023; 13:e073716. doi: 10.1136/bmjopen-2023-073716

Probes:

- ***"unemployment"*** → *How does not having a job affect your and your babies' nutrition?*
- *"**expensive** food"* → *Are there other any other ways you could get nutritious food?*
  → *What food do moms have if they can't get healthy food?*
- ***"no healthy stores"*** → *what makes it difficult to access supermarkets?*
- ***"little grant money"*** → *You mentioned grants. Let me ask about a scenario when two poor mothers both receive the child support grant. For one of the mothers the grant lasts longer. For the other if finishes in a week. Can you think of the reasons why?*

How does *[e.g. difficulty to get to supermarkets; everyone selling vetkoeks; eating only pap and rice/vetkoek]* affect moms ability to follow the nutrition advice?

What else makes it difficult for a mother to **buy** nutritious and healthy diet?

What makes it difficult for a mother to **prepare** nutritious and healthy food?

What do others think about *[these needs/concerns]?*

PART 2 – LOCAL & COMMUNITY SOLUTIONS

Okay. We talked about a number of challenges, such as *(summarize what participants said)* let's focus more on local solutions. **We all know that the government could and should do more. But government solutions takes a long time.** While we are waiting for the government, **we need quick local solutions.**

We'll go around the table asking each person to pick a challenge we talked about and recommend an existing or imagined service to overcome the challenge.

Any questions before we start? *PAUSE for questions. Pick a person to start.*
Let's begin with you.

QUESTION 4:
You mentioned *(pick a challenge, e.g., difficult to go to the supermarket)* - what would be the solution to that?

Probes:

- ***"government should"*** → Besides the government solution, what can we do in the community?
- ***"more jobs"*** → Besides the government creating jobs, what can be done in the community? Do you know of mothers who were unemployed and now have jobs?
- ***"more grant money"*** → *You mentioned that often people don't know how to maintain the budget. Would more grant money be spent more wisely?*
- ***"More income"*** → *can you think of community initiatives to overcome financial issues?*

- ***"Less expensive food"*** *→ are there other ways to cut our spending on food in the shops?*

QUESTION 5: Follow up to mentioned solutions:

- **Do you know of any solution like that which exists in your community?**
- **Who should deliver this solution?**
- **What would it take to make this solution a reality?**
- **How does this solution help moms to ensure nutritious food?**

What do other people think? What else can be done?

QUESTION 6:

To what extent moms participate in community groups or community-based organization that can help address their challenge?

PART 3 - Closing the FGD

Once everyone has had a turn, ask the group if anyone wants to suggest other challenges, if they are satisfied with the solutions discussed.

- Have we covered the most important challenges around nutrition?
- How satisfied are you with the recommendations?
- Who would like to make any other recommendations?

Okay. I hope you are happy with the solutions and that you enjoyed this exercise. Thank you for your important insights. We hope to discuss your perspectives with your local stakeholders in the future. Thank you for your time. Before you leave, please go to my colleague, to collect travel reimbursement money and sign a registry.

Supplemental file 4 - Coding structure

Code	Description
**Theme: Available services and resources**	
Food aid	Food parcels and food vouchers distributed by the Department of Social Development, community-based organizations, churches, schools, and clinics.
Crèches	Childhood education centres that offer childcare services, including feeding schemes.
Stokvels	An invitation-only group of twelve or more people serving as a rotating credit union or saving scheme.
Skills training	Any learnership, fellowship programmes, volunteering opportunities in the community.
Clinic-based services	Services including nutrition advice, dietitian consultations, antenatal and postnatal care, supplementation, and social work support provided within clinical settings.
Poverty alleviation projects	Community projects such as knitting, sewing, and beading projects, baking groups and co-operatives in the community.
Food garden	Refers to food gardens located at schools, community-based organizations, and within households.

Social grants	Includes reference to the South African Child Support Grant, Disability Grant, Old Age Grant, Foster Child Grant, Care Dependency Grant, and Social Relief of Distress Grant.
**Theme: sources of variation**	
Delayed/periodic access to healthier food	Circumstances of delayed social grant payment, temporary access to healthier food upon income or social grant receipt. Includes references to running out of food budget by the end of the month.
Perceived and experienced mistrust, corruption, exclusion	Perceptions around unfairness in food aid distribution, local job recruitment including exclusion from food gardens due to young age and from economic opportunities due to old age.
Life skills	Refers to digital, technological, and internet literacy; challenges with CV writing, job search and application, and form completions.
Ability to pay for transportation, printing, internet	Transportation costs associated with access to supermarkets, government departments, skills training, and job opportunities.
School dropouts	Refers to dropping out of school often due to inadequate financial and childcare support for expectant and new mothers, and lack of necessary documentation for receipt of matric certificate.
Service eligibility	Possession of matric certificate, birth certificate, identification document.
Poor living conditions	Inadequate garden space, refrigeration, storage space and lack of stable electricity.
Household's food budget must cater for all vs individuals' need	Challenge of managing limited resources to provide food that satisfies everyone in the household while considering diverse nutritional needs of pregnant and lactating mothers, and young children.
Overcrowding, sharing of scarce resources	Refers to households with many members in limited space, leading to shorter durations of benefit from services like food parcels and vouchers, as resources must be divided among more people.
Competing household expenses	Refers to cases when healthier foods are sacrificed as budget is needed for monthly rent payment, school-related expenses (e.g., uniforms, stationary), maternity and baby clothing, toiletries, and pampers.
Lack of control over nutrition in the household	Refers to the inadequate control of mothers and pregnant women over household's nutrition budget and limited say in household food selection.
Grant-, kinship-, loan-dependency	Financial and livelihood dependency on income of household members, partners, elderly, loans, unfavourable relationships.
Single motherhood/absent fathers	Refers to cases where fathers are not actively engaged in providing material or emotional support to mothers and their children. Includes references to significant implications for the well-being and financial stability of mothers and children.
Poor partner and family dynamics	Represents a complex set of challenges related to familial and interpersonal relationships, including rejection, conflicts, and unsupportive behaviours.
Know-how of eating healthy on a budget	The knowledge and skills required to maintain a nutritious diet while managing limited financial resources. It includes strategies such as using savings to incorporate variety into one's diet, preparing meals at home to save money, seeking discounts and deals when shopping, and making cost-effective purchases by buying items in bulk.

Awareness of healthier diet	Refers to a combination of factors, including familiarity with a varied and balanced diet, understanding of nutrition beyond fruits and vegetables, willingness, and desire to incorporate variety into one's dietary.
Ability to trade off present investment for future return	Refers to financial investment (e.g., stokvels) and time investment (e.g., skills training).
Healthcare and welfare stigma	Encompasses the subjective sense of experiencing negative attitudes or societal judgment in situations involving healthcare interactions, teenage pregnancy, or seeking help from the community.
Ability to save, maintain and multiply budget	Refers to a range of skills and strategies, including budgeting expertise, income generation through garden production and entrepreneurship, and participation in community savings groups like stokvels. It reflects the capability to effectively manage and grow financial resources through various means.
Poor coping (mental health and substance abuse)	reflects the struggle to effectively manage stress and life difficulties, resulting in negative mental health outcomes and engagement in harmful behaviours like debt accumulation, gambling, and substance abuse.
Low self-esteem and hopelessness	Feelings of being stuck, sense of hopelessness, and a lack of motivation, seeing a “way out”. In the context of motherhood, it refers to a lack of knowledge about pregnancy and parenting, leading to negative influences on how mothers perceive their own capabilities and effectiveness in their parenting role.
Domestic violence	This includes child neglect, gender-based violence, financial, emotional abuse within the household.
Influence of local food environment	Rerefers to choosing unhealthy food over healthy due to cost, the abundance of convenient but unhealthy produce, and the lack of locally available fresh and healthy alternatives. Includes reference to long distance from supermarkets, compromising quality and quantity of food purchased.
Social connectedness	Reflects the level of involvement and connection of mothers and pregnant women within their social environment. Includes engagement in self-help groups, awareness of community resources and services, participation in savings clubs (stokvels), and church groups.
Lack of belonging, isolation	Refers to the experience of feeling disconnected, alone, or unsupported (financially and emotionally) in the role of motherhood. It reflects the absence of a supportive peer network and emotional closeness, contributing to a sense of isolation and detachment.
**Theme: Social needs**	
Affordable healthier foods	Links to theme 2 codes: Delayed/periodic access to healthier food, Ability to pay for transport to supermarkets, Awareness of healthier diet (beyond fruits and vegetables i.e., legumes, brown rice), Influence of local food environment.
Social support	Includes psychosocial support that links to theme 2 codes: Healthcare and welfare stigma, Low self-esteem and hopelessness, Poor coping (mental health and substance abuse), Poor partner and family dynamics, Domestic violence.<br>Further, it includes social cohesion and connectedness that links to theme 2 codes: Perceived and experienced mistrust, corruption, exclusion, Lack of belonging, isolation, Social connectedness (i.e., group membership).
Access to civic services, welfare, and labour market	Service eligibility (i.e., possession of identification documents, birth certificate, matric certificate); School dropouts; Life skills (i.e., internet

	literacy, CV writing, form completion); Ability to pay for transportation, printing, internet
Appropriate and affordable housing	Links to theme 2 codes: Monthly rent payments; Overcrowding, sharing of scarce resources; Household's food budget must cater for all vs individuals' nutritional needs; Poor living conditions (i.e., lack of stable electricity, storage space, refrigeration)
Personal income stability	Links to theme 2 codes: single motherhood/absent fathers, Grant-, kinship-, loan-dependency, Lack of control over nutrition in the household.
Financial planning for nutrition	Links to theme 2 codes: Ability to save, maintain and multiply budget; Ability to trade off present investment for future return; Know-how of eating healthy on a budget (i.e., buying in bulk, home preparation)

Supplemental file 5 - Consolidated criteria for reporting qualitative studies (COREQ): 32-item checklist

Developed from: Tong A, Sainsbury P, Craig J. (2007) Consolidated criteria for reporting qualitative research (COREQ): a 32- item checklist for interviews and focus groups. International Journal for Quality in Healthcare: 19:349 – 357

**No. Item**	**Guide questions/description**	**Reported on Page #**
**Domain 1: Research team and reflexivity**		
Personal Characteristics		
1. Interviewer/facilitator	Which author/s conducted the interview or focus group?<br><br>*Interviews were conducted by AE, and SN (see in acknowledgements) facilitated the focus groups.*	7
2. Credentials	What were the researcher's credentials? E.g. PhD, MD<br><br>*AE – MSc*<br>*SN - Matric exemption certificate*	N/A
3. Occupation	What was their occupation at the time of the study?<br><br>*AE – Senior Researcher*<br>*SN – Qualitative research assistant*	N/A
4. Gender	Was the researcher male or female?<br><br>*Both interviewers identified as women.*	N/A
5. Experience and training	What experience or training did the researcher have?<br><br>*AE and SN had experience in qualitative interviewing and working with members of the community in the study setting.*	7
Relationship with participants		
6. Relationship established	Was a relationship established prior to study commencement?<br><br>*The AE and SN were unknown to participants prior to the commencement of the study and recruitment.*	N/A

7. Participant knowledge of the interviewer	What did the participants know about the researcher? e.g. personal goals, reasons for doing the research<br><br>*AE and SN introduced themselves at beginning of the interviews and focus groups, explained their roles, occupations, and purpose of the research.*	Supplemental file 2 and 3
8. Interviewer characteristics	What characteristics were reported about the inter viewer/facilitator? e.g. Bias, assumptions, reasons and interests in the research topic<br><br>*See above*.	Supplemental file 2 and 3
**Domain 2: Study design**		
Theoretical framework		
9. Methodological orientation and Theory	What methodological orientation was stated to underpin the study? e.g. grounded theory, discourse analysis, ethnography, phenomenology, content analysis<br><br>*A novel theoretical framework was developed for the study that informed data collection and analysis. Thematic analysis was used to analyse the data.*	4-6
Participant selection		
10. Sampling	How were participants selected? e.g. purposive, convenience, consecutive, snowball<br><br>*Purposive sampling.*	6
11. Method of approach	How were participants approached? e.g. face-to-face, telephone, mail, email<br><br>*To approach interview participants, an invitation letter was sent to their respective community-based organization, followed up with a telephone call. Focus group discussion participants were approach in person at health care facilities.*	6
12. Sample size	How many participants were in the study?<br><br>*18 employees representing 10 community-based organization were interviewed, and 51 pregnant women and mothers participated in focus groups.*	8
13. Non-participation	How many people refused to participate or dropped out? Reasons?<br><br>*None*.	N/A
Setting		
14. Setting of data collection	Where was the data collected? E.g. home, clinic, workplace<br><br>*The interviews were conducted either in person at the community-based organization or via telephone. Focus groups took place at a research centre in the community.*	7
15. Presence of non-participants	Was anyone else present besides the participants and researchers?	N/A

	No.	
16. Description of sample	What are the important characteristics of the sample? e.g. demographic data, date<br><br>*Please refer to Table 2.*	8 and Table 2
Data collection		
17. Interview guide	Were questions, prompts, guides provided by the authors? Was it pilot tested?<br><br>*Yes, please refer to supplemental file 2 and 3.*	6 and supplemental file 2 and 3
18. Repeat interviews	Were repeat interviews carried out? If yes, how many?<br><br>*No.*	N/A
19. Audio/visual recording	Did the research use audio or visual recording to collect the data?<br><br>*Yes, the interviews and focus groups were audio-recorded with consent from participants.*	6
20. Field notes	Were field notes made during and/or after the interview or focus group?<br><br>*Memos in the form of digital notes were taken during the data analysis process to help with theme generation and refinement.*	N/A
21. Duration	What was the duration of the inter views or focus group?<br>*The interviews and focus groups lasted about an hour.*	7
22. Data saturation	Was data saturation discussed?<br><br>*Transcripts were analysed progressively to determine the point at which saturation was achieved.*	7
23. Transcripts returned	Were transcripts returned to participants for comment and/or correction?<br><br>*No.*	N/A
**Domain 3: analysis and findings**		
Data analysis		
24. Number of data coders	How many data coders coded the data?<br><br>*One (AE).*	7
25. Description of the coding tree	Did authors provide a description of the coding tree?<br><br>*Yes, please refer to supplemental file 4.*	7 and supplemental file 4
26. Derivation of themes	Were themes identified in advance or derived from the data?<br><br>*Themes were identified through a hybrid (deductive and inductive) approach.*	7
27. Software	What software, if applicable, was used to manage the data?	7

	MAXQDA 2022 data analysis software.	
28. Participant checking	Did participants provide feedback on the findings?<br><br>*A summary of the findings was presented to members of the same community in the next phase of the bigger research projects. Community members (different from the present study's participants) further engaged with the findings and informed the larger study's objectives.*	N/A
Reporting		
29. Quotations presented	Were participant quotations presented to illustrate the themes/findings? Was each quotation identified? e.g. participant number<br><br>*Yes, interview participant quotations were identified by gender, role, and organization number. Focus group participants were identified by pregnancy status and focus group number. All findings were illustrated with relevant quotations.*	11-14
30. Data and findings consistent	Was there consistency between the data presented and the findings?<br><br>*Yes, all data was interpreted in the Discussion section and was linked to existing literature.*	14-16
31. Clarity of major themes	Were major themes clearly presented in the findings?<br><br>*Yes.*	9-13
32. Clarity of minor themes	Is there a description of diverse cases or discussion of minor themes?<br><br>*Minor themes were discussed as part of each major theme. We identified only minor instances of differing perspectives amongst participants. These were reported as part of the relevant themes. Please refer to page 13 for an example.*	13

Erzse A, *et al. BMJ Open* 2023; 13:e073716. doi: 10.1136/bmjopen-2023-073716
