# Supplementary material for: Qualitative exploration of the constraints on mothers’ and pregnant women’s ability to turn available services into nutrition benefits in a low-resource urban setting, South Africa
Source: BMJ Open. 2023 Nov 22;13(11):e073716. doi: 10.1136/bmjopen-2023-073716 (PMC10668265; doi:10.1136/bmjopen-2023-073716)
Supplement: Author's manuscript [file bmjopen-2023-073716.draft_revisions.pdf]

BMJ Open

BMJ Open is committed to open peer review. As part of this commitment we make the peer review history of every article we publish publicly available.

When an article is published we post the peer reviewers' comments and the authors' responses online. We also post the versions of the paper that were used during peer review. These are the versions that the peer review comments apply to.

The versions of the paper that follow are the versions that were submitted during the peer review process. They are not the versions of record or the final published versions. They should not be cited or distributed as the published version of this manuscript.

BMJ Open is an open access journal and the full, final, typeset and author-corrected version of record of the manuscript is available on our site with no access controls, subscription charges or pay-per-view fees (http://bmjopen.bmj.com).

If you have any questions on BMJ Open's open peer review process please email info.bmjopen@bmj.com

BMJ Open

Variations in mothers' and pregnant women's capacity to benefit from the service environment to enhance nutrition in a low resource urban setting, South Africa: a qualitative exploration

Journal:	*BMJ Open*
Manuscript ID	bmjopen-2023-073716
Article Type:	Original research
Date Submitted by the Author:	15-Mar-2023
Complete List of Authors:	Erzse, Agnes ; University of the Witwatersrand Johannesburg Faculty of Health Sciences, SAMRC/Centre for Health Economics and Decision Science- PRICELESS SA<br>Desmond, Chris; University of the Witwatersrand Johannesburg Faculty of Health Sciences, SAMRC/Centre for Health Economics and Decision Science- PRICELESS SA<br>Hofman, Karen; University of the Witwatersrand Johannesburg Faculty of Health Sciences, SAMRC/Centre for Health Economics and Decision Science- PRICELESS SA<br>Barker, Mary; University of Southampton Faculty of Environmental and Life Sciences, School of Health Sciences; University of Southampton Faculty of Health Sciences, MRC Lifecourse Epidemiology Centre<br>Christofides, Nicola; University of the Witwatersrand Johannesburg Faculty of Health Sciences
Keywords:	Health Equity, Health policy < HEALTH SERVICES ADMINISTRATION & MANAGEMENT, Community child health < PAEDIATRICS, PUBLIC HEALTH, QUALITATIVE RESEARCH

SCHOLARONE™
Manuscripts

BMJ

I, the Submitting Author has the right to grant and does grant on behalf of all authors of the Work (as defined in the below author licence), an exclusive licence and/or a non-exclusive licence for contributions from authors who are: i) UK Crown employees; ii) where BMJ has agreed a CC-BY licence shall apply, and/or iii) in accordance with the terms applicable for US Federal Government officers or employees acting as part of their official duties; on a worldwide, perpetual, irrevocable, royalty-free basis to BMJ Publishing Group Ltd ("BMJ") its licensees and where the relevant Journal is co-owned by BMJ to the co-owners of the Journal, to publish the Work in this journal and any other BMJ products and to exploit all rights, as set out in our licence.

The Submitting Author accepts and understands that any supply made under these terms is made by BMJ to the Submitting Author unless you are acting as an employee on behalf of your employer or a postgraduate student of an affiliated institution which is paying any applicable article publishing charge ("APC") for Open Access articles. Where the Submitting Author wishes to make the Work available on an Open Access basis (and intends to pay the relevant APC), the terms of reuse of such Open Access shall be governed by a Creative Commons licence – details of these licences and which Creative Commons licence will apply to this Work are set out in our licence referred to above.

Other than as permitted in any relevant BMJ Author's Self Archiving Policies, I confirm this Work has not been accepted for publication elsewhere, is not being considered for publication elsewhere and does not duplicate material already published. I confirm all authors consent to publication of this Work and authorise the granting of this licence.

For peer review only

Title: Variations in mothers' and pregnant women's capacity to benefit from the service environment to enhance nutrition in a low resource urban setting, South Africa: a qualitative exploration

Authors: Agnes Erzse[1*], Chris Desmond[1], Karen Hofman[1], Mary Barker[2,3,4], Nicola Christofides[4]

[1]SAMRC/Wits Centre for Health Economics and Decision Science—PRICELESS SA, School of Public Health, Faculty of Health Sciences, University of Witwatersrand, Johannesburg, South Africa
[2] School of Health Sciences, Faculty of Environmental and Life Sciences, University of Southampton, Southampton, UK
[3]MRC Lifecourse Epidemiology Centre, Faculty of Medicine, University of Southampton, UK
[4]Faculty of Health Sciences, University of the Witwatersrand, Johannesburg, South Africa

Corresponding author: Agnes Erzse, agnes.erzse@wits.ac.za, +27715472158, 27 St. Andrews Road, Parktown, Johannesburg 2193, Republic of South Africa

Key words: maternal and child health, social need, nutrition, inequality, capabilities approach, South Africa

Abstract

Introduction: Inequalities in child and maternal malnutrition occur across the globe, including in South Africa. Among the most vulnerable are mothers and children living in urban settlements characterized by multidimensional poverty. Mothers in this context may need different types and quantities of services and resources to achieve optimal nutrition. This exploratory qualitative study explores existing services perceived as important to nutrition, variations in mothers' capacity to benefit from these services and in the unmet needs driving these variations between women.

Methods: Between February and October 2022, seven focus groups were conducted with 51 mothers including 21 pregnant women, and 18 semi-structured interviews were conducted with employees from 10 community-based organizations in Soweto in Johannesburg. Focus groups and interviews were audio-recorded and transcribed verbatim. Data was analysed thematically using a novel social needs framework developed for this study that combines an ecological with a capabilities approach. The study defines social needs as the requisites that can magnify (if unmet) or reduce (if met) variation in the degree to which individuals can benefit from existing services.

Results: Mothers identified social needs related to financial planning, personal income stability, appropriate and affordable housing, access to government services, social support, and affordable healthier foods. The degree to which these needs were met determined mothers' capabilities to benefit from eight services. These were clinic-based services including nutrition advice and social work support, social grants, food aid, community savings groups, poverty alleviation projects, skills training workshops, formal employment opportunities and crèches/school feeding schemes.

Conclusion: Findings demonstrate that while current social protection mechanisms and free health services are necessary, they are not sufficient to address nutrition inequalities. Women's social needs must also be met to ensure that existing services are accessed and used to improve the nutrition of all mothers and their children.

Key messages

What is already known on this topic?

- Despite evidence of effective healthcare services and social protections, nutritional inequalities occur across and within countries, including South Africa.

For peer review only - http://bmjopen.bmj.com/site/about/guidelines.xhtml

- Current approaches to understanding and addressing nutrition inequalities are limited in vulnerable populations.
- New approaches are needed that consider the variation in individuals' ability to benefit from available service environments.

What this study adds?

- A novel social needs approach to improving how we research and design strategies to address nutrition inequalities.
- Practical application of the framework to identify social needs that limit mothers' capacity to benefit from services in a resource constrained urban township.

How this study might affect research, practice or policy?

- Identifying and addressing social needs will enhance the potential of available healthcare services and social protection to produce optimal and equal nutrition outcomes.
- Additional strategies are necessary to meet social needs across multiple sectors to enhance uptake of services and so reduce nutrition inequities.

Introduction

Poor maternal and child nutrition is one of the biggest preventable causes of ill health and poor human development globally. Despite its commitment to the 2025 global nutrition targets set by the World Health Assembly (1), South Africa is not on track to achieve a 30% reduction in low birthweight and 40% in stunting. Over the past decade, no progress has been made with low birthweight remaining the same; from the benchmark of 14.8% and current prevalence of 15% (2). Stunting among under five years has risen from 23% in 2012, to 27% of children under five being stunted in 2016 (3). Moreover, under five childhood overweight and obesity rates are essentially unchanged (4). Similarly, no progress has been made achieving a 50% reduction in anaemia in women from 28.6% in 2012, with current figures being at 30% (5) and 33% of pregnant women are obese or overweight and 9% have gestational diabetes (6).

There are significant inequalities in nutrition outcomes across and within countries. Disparities are particularly evident in South Africa. Among those at the bottom end of the distribution are mothers and children in lower-income urban townships, have worse nutrition outcomes than the national average. Soweto for example, the largest township in South Africa, has more low birthweight babies (1.6 percentage points), more stunted and obese children under the age of five (7 and 11 percentage points respectively) (7), and more obese mothers (33%) (8).

These disparities exist despite free maternal and child health care; the presence of high impact first 1000 days interventions (9), and social protection mechanisms such as the Child Support Grant (ZAR 450 or $26/month as of 2023) (10). This suggests that the availability of universal primary care services may not be enough to ensure good nutrition for all mothers and their children. Families may need to access different types and amounts of services and resources such as policies, foods, changes in social norms, and infrastructure, to achieve the same level of optimal nutrition. This variation challenges the way we think about prioritizing and investing in interventions for nutrition. Both globally and in South Africa, the focus has been on increasing the coverage of individual level curative and population level preventative nutrition programmes (11–13). Less consideration has been given to what makes it possible for individuals to benefit from these existing services.

If nutrition is influenced by variations in needs and opportunities mothers' and pregnant women face, a new theoretical approach that embraces this complexity is necessary. The aim of this paper is to examine these variations through engagement with key stakeholders (mothers, pregnant women, and

For peer review only - http://bmjopen.bmj.com/site/about/guidelines.xhtml

community-based organizations) in Soweto and to operationalize a novel conceptual framework combining ecological and capabilities approaches.

Existing theoretical approaches: missing social needs?

Bronfenbrenner (1977) was one of the earliest theorists to capture the complexities of human development through his ecological model comprised of an arrangement of nested circular systems (14). His model posits that an individual's development is the result of the dynamic interplay among multiple systems of influence, including the micro- (inner-most circle), meso-, exo-, and macrosystem outer-most circle). A fundamental principle that sets the micro- and mesosystem apart from the exo- and macrosystems is the extent to which these reflect individual experience. Bronfenbrenner envisioned that the micro- and mesosystems refer to the face-to-face interaction of individuals with the immediate settings such as home, neighbourhood, and social networks. By contrast, the exo- and macrosystem consist of social structures (e.g., institutions like government agencies) that regulate individuals' lives at the meso and micro level yet are removed from the individual (15). Bronfenbrenner's micro, meso, and macro levels have become the foundation for common typologies in the public health literature to describe the drivers of, and strategies to improve, nutrition (16). Table 1 summarizes some of the most used typologies in the nutrition literature as they relate to the ecological levels.

Table 1. Overview of common typologies used to describe drivers of and interventions for nutrition with examples

Ecological model	Determinants	Streams	Specificity
Micro level	Nutrition needs (i.e., iron deficiency)	Downstream (i.e., iron supplementation)	Nutrition specific
Meso level	Social needs (i.e., transportation, housing)	Mid-stream (i.e., community-based services)	Nutrition sensitive
Macro level	Structural/social determinants of health (i.e., poverty)	Upstream (i.e., social safety nets)	

At the micro level, there are immediate determinants of nutrition (i.e., iron deficiency) which are commonly addressed through medical, nutrition specific actions (i.e., iron supplementation) (17). Using the metaphor of a stream (18), these actions can be described as downstream (19). By contrast, upstream actions are located at the macro level and are viewed as nutrition-sensitive (17) as they address the underlying structural and social determinants of health, such as poverty and unemployment. Interventions addressing social determinants of health range from education policies, taxes on unhealthy foods to interventions to improve women's status in society (19), all of which do not involve the individual directly.

Notwithstanding the need for services at the micro and macro levels to improve nutrition, the inequalities described earlier suggest a need to identify meso level strategies that address sources of variation in individuals' capability to benefit from existing services. Sources of variation could include personal (i.e., possession of identity documents) and socio-economic factors (i.e., cost of healthy food), that can lead to avoidable variation in capability to benefit from services if the associated need – referred to as social need – is not met. In this context, social needs are the requisites that can magnify (if unmet) or reduce (if met) the sources of variation individuals face when benefiting existing services. As such, variations in social needs influence the effects of both macro and micro level services on nutrition and may therefore be responsible for consequent disparities in nutrition outcomes.

For peer review only - http://bmjopen.bmj.com/site/about/guidelines.xhtml

There is a clear overlap between social needs and “conversion factors”, a concept central to Amartya Sen’s “capability approach” (20). The capability approach focuses on the conversion of resources into individual capabilities (real opportunities) to achieve a valuable combination of human functionings, termed "beings" and "doings". From a nutrition point of view, resources can be services and "beings" and "doings" can include being well-nourished. The capabilities approach recognizes that there are personal, social and environmental aspects of the environment (conversion factors) that either expand or restrict one’s real opportunities (capabilities) to convert resources into valued functionings (21). Similarly, social needs play key roles in determining the degree to which mothers can benefit from government and healthcare services for optimal nutrition. The theoretical complementarity between the ecological model and the capabilities approach provides reason to believe that the integration of the two could help better understand what mothers in their communities need so that they are capable of converting available government and healthcare services into better nutrition outcomes.

A social needs framework

The two models combined (Figure 1) allow for mothers’ diversity of needs and capabilities to benefit from existing services. Furthermore, it reflects the interaction between ecological levels by demonstrating how such diversity at the meso level determines the effect of an existing service environment on nutrition.

[place of Figure 1]

Figure 1. A social needs framework combining the capabilities approach (20) and the ecological model (22)

The framework groups all services into a combined service environment at the macro level based on the assumption that a “service is a service” for an individual regardless of whether the service targets downstream or upstream determinants. What the model seeks to highlight is that the availability of a service is not an end in itself. The effect of the service (indicated with the arrow on Figure 1) is determined by the conversion factor as it passes through the meso level that determines mothers’ real opportunities (capabilities) to benefit from services. This reflects how mothers’ experiences of a given service environment and the degree to which they can convert services into nutrition benefits, varies. One might fully benefit from a service by accessing and implementing it, another might partially benefit by accessing it but not being able to use the benefits it offers, and a third might not be able to access or benefit from the service. Where an individual sits on this benefit scale is determined by their social needs and the degree to which these social needs are met. In this sense, the framework identifies conversion factors as the product of social needs.

It is important to note that not all services will see variation in mothers’ capability to benefit (conversion factors), and not all variation will be influenced by the same social needs. By applying this framework, the paper aims to explore which services (either macro or micro) see big variation in mothers’ conversion factors (capability to benefit) and identify some of the social needs that shape these conversion factors. This framework is particularly relevant in dealing with the nutrition of disadvantaged people or of socioeconomic groups in unfavourable circumstances or conditions because they are likely to have multiple unmet social needs.

One approach to increasing understanding of social needs is to engage those who have direct experience of these needs. These include mothers and pregnant women, as well as community-based organizations (CBOs), defined as any non-profit organization operating at meso levels in community

For peer review only - http://bmjopen.bmj.com/site/about/guidelines.xhtml

settings. The rationale behind engaging CBOs is that they interact and monitor community members and are therefore able to identify their unique challenges.

In a context of available macro level government and micro level nutrition services in Soweto, Johannesburg, this research sought to understand 1) existing services which are perceived as valuable for nutrition but that not all mothers can benefit from equally, 2) the sources of variation in mothers' capability to benefit from those services, and 3) associated social needs that if met could reduce this variation.

Methods

This cross-sectional, exploratory qualitative study was conducted in Soweto between February and October 2022. It involved semi-structured individual interviews (interviews) with employees from CBOs and focus group discussions (FGD) with mothers and pregnant women (18-49 years) attending antenatal and post-natal clinics.

Setting

Soweto is a densely populated working class township which by 2019 contained about 1.8 million people, one third of Johannesburg's population (23). Inhabitants are almost entirely black South Africans and 80% of adults are unmarried (24). Forty percent of households are headed by women and one in two children grow up without their father in the home (25). In some parts of Soweto, individuals endure household and community violence, crime and extreme poverty with 19% of all households not receiving any income (24). Lower income areas are characterized by unpaved roads, lack of drainage systems, houses with tin roofs, shared drinking water sources and toilet facilities (26,27). Cohabitation of several families within a four roomed government house is a common feature, with many accommodated in smaller rooms or shacks built at the back of the house (26). A 2018 survey found that young mothers in Soweto are particularly vulnerable to poor-quality housing, experience lower levels of education and high rates of unemployment (64%) (27). Compared to nulliparous women, young mothers are also more likely to face food insecurity and have poorer health outcomes, including higher levels of overweight and obesity (27).

Sampling

The selection of interview participants began by developing a contact list of Soweto-based CBOs from an internet search, including two CBO directories (28,29). CBOs were identified as eligible if the mission/vision statement and overview of services contained explicit language around any of the following: provision of maternal and child health services, social support and nutrition to children, mothers, and pregnant women. CBOs focusing primarily on older persons, sick people (i.e., HIV and AIDs, TB), disabled people, crime prevention and drug abuse were excluded. Fifteen CBOs were contacted, and 10 were included in the final sample. An invitation letter was sent to CBOs, followed up with a telephone call. Potential participants put forth by the CBOs were approached by the first author and asked if they were willing to participate in the study.

FGD participants included purposively sampled pregnant women attending antenatal care and mothers attending postnatal care at five primary health care facilities in Soweto (Appendix A). Recruitment was undertaken by a local multi-lingual qualitative researcher with experience of working with mothers in Soweto. The researcher approached individuals in communal areas of the health care facilities (e.g., the main corridor, waiting areas) and invited those interested if they resided in Soweto and were 18 years of age and above. The sample size for both the interviews and FGDs was determined by data saturation (30).

Data collection

For peer review only - http://bmjopen.bmj.com/site/about/guidelines.xhtml

An interview and a FGD guide were developed separately to assist data collection. Guides asked participants about their perceptions of the challenges that affected maternal and child nutrition in the community, as well as the available resources, or services that they knew of and might help addressing these challenges. The interviews were conducted in English by the first author, either in person at the CBO or via telephone. The FGDs were facilitated by a trained, multi-lingual researcher, familiar with customs and traditions of the community. FGDs participants expressed themselves in English and in their vernacular languages (Xhosa, Zulu, or Sotho). FGDs were organized after the completion of the interviews, and these took place at a research centre in Soweto. All FGD participants completed a sociodemographic questionnaire. The feasibility of our data collection tools was verified through a pilot interview and FGD. Results from the pilots were included in the analysis. The interviews and FGDs lasted about an hour and all participants were reimbursed for time and transport. Written consent was taken from all participants, and the study protocol was granted ethical approval by the University of Witwatersrand Ethics Committee (M210718) and the Research Committee of Johannesburg Health District (GP_202110_002).

Public involvement

The data collection followed an iterative process, whereby interviews with CBOs informed the development of the FGD guide. Further, early FGDs resulted in adjustments to subsequent ones to enhance the relevance of the questions. A summary of the findings will be presented for validation to participants and patients (attending antenatal and postnatal care).

Data management and analysis

Interviews and FGDs were digitally recorded, transcribed, and translated into English where necessary. The first author used a hybrid (deductive and inductive) approach to build the coding structure and coded the interviews and FGD transcripts with MAXQDA 2022 data analysis software (31) using thematic analysis (32). Data was triangulated from the interviews and FGDs to identify similarities and differences and looked at convergence patterns to validate interpretation. Once inductive and deductive codes were developed, codes were organized into broader categories based on thematic similarities. Lastly, categories were re-organized by three deductive themes anchored in our study objectives and framework (Figure 1): services valuable for nutrition (but which not all mothers could benefit from), sources of variation, and social needs. The co-authors discussed their interpretations of the data. Verbatim quotes from participants have been selected to illustrate how FGD participants and CBOs accounts were linked to themes. Reporting of the findings adheres to COREQ guidelines (33).

Results

We present the characteristics of participants then discuss the six social needs identified by the participants as they link to multiple sources of variation in mothers' and pregnant women's (hereinafter mothers) capability to benefit from eight different services that were identified as valuable for optimal maternal and child nutrition (hereinafter nutrition).

Characteristics of CBO participants

We interviewed 18 employees representing 10 CBOs that serviced clients in 11 of 40 Soweto suburbs. Eight CBOs aimed to directly improve the well-being of orphans and vulnerable children and adolescents (0-18 years of age), and two CBOs aimed to directly improve the well-being of women, including mothers and pregnant women. On average, there were 2 participants per CBO. All but one participant were women, and they represented Founders and Directors (n=4), managers responsible for directing programs and employees (n=2), social workers (n=11) and a volunteer (n=1) who worked directly with beneficiaries.

Characteristics of FGD participants

For peer review only - http://bmjopen.bmj.com/site/about/guidelines.xhtml

Fifty-one women participated in 7 FGDs (see group compositions in Appendix A). Twenty-one (41%) were pregnant (12 of them for the first time) and of the 39 non-first-time mothers (71%) had at least 2 children. Twenty-nine (56%) participants were single while 17 (41%) were partnered or married. All but two participants were unemployed, and the majority relied on social grants (64%) as their main source of income. Thirty-five participants (68%) reported their household income to be less than ZAR 3000 (USD 187) per month, 77% being less than R1000 (USD 57.94). Mothers' living conditions differed. Some lived in shacks/informal dwellings (31%), others in owner-occupied houses (bond house) (21%), government subsidised houses (21%), or single rooms built in the backyard of other households (19%). Thirty-two mothers (62%) had stable electricity, 38 (74%) had a fridge, and 10 (19%) had daily access to internet. On average there were 5 people living in their houses (range 2-11). Less than half (45%) reported being members of community groups, and 10 (43%) of those who did were part of *stokvels* (community money savings groups). Details of FGD participant are provided in Table 2.

Table 2. Sociodemographic characteristics of FGD participants

**Age (years)**		
Minimum-maximum; Mean [SD]	18-49	31 [8]
**Pregnancy status**	**N**	**%**
Pregnant	21	41.1
Non-pregnant	30	58.8
**Number of babies/children cared for**		
0 - first time mother	12	23.5
1	11	21.5
2-4	25	49.0
5 and above	3	5.8
**Marital status**		
Single never married	29	56.8
Married	4	7.8
Partnered	17	33.3
Separated	1	1.9
**Highest level of schooling**		
Primary school	2	3.9
Some high school	20	39.2
Completed high school	25	49.0
Diploma/higher diploma	4	7.8
**Household income**		
ZAR 1000 or less	27	52.9
ZAR 1001- 3000	8	15.6
ZAR 3001-R5000	10	19.6
ZAR 5001-R10000	3	1.9
ZAR 10001-20000	3	5.8
**Source of income**		
Government grants	33	64.7
Formal employment	2	3.9
Informal employment	2	3.9
Others in the household	8	15.6
**Type of house**		

For peer review only

Bond house	11	21.5
Government subsidised houses	11	21.5
Shack or informal dwelling	16	31.3
Single outside room	10	19.6
Other	2	3.9
**Number of people in the house**		
Minimum-maximum; Mean [SD]	1-11	5 [2]
**Household assets**		
Stable electricity	32	62.7
Radio	33	64.7
A fridge	38	74.5
Television	38	74.5
Cell phone	33	64.7
Daily access to internet	10	19.6
Car	4	7.8
Clothing sufficient to keep mothers and children warm	38	74.5
**Membership**		
Stokvel	10	19.6
Community garden group	2	3.9
Mothers support group	2	3.9
Women's association	2	3.9
Community service (school development committee)	1	1.9
Religious groups	3	5.8
Any	23	45.0

Social needs of mothers

The analysis identified six social needs: financial planning; personal income stability; appropriate and affordable housing; access to civic services, welfare, and the labour market; social support; and affordable healthier foods. The following section reports on each of these needs as they link to multiple sources of variation that enabled or disabled mothers from benefitting from eight different services. These services were recognized as valuable for nutrition but there was variation in mothers' capability to benefit from them. These included clinic-based services (nutrition advice, social workers); social grants (Child Support Grant, Social Distress Relief); food aid including parcels and vouchers (distributed by the Department of Social Development, churches, CBOs, crèches/schools and clinics); stokvels; poverty alleviation projects (sewing, baking groups and food gardens at schools, and CBOs); skills training workshops; formal employment opportunities including learnerships; and crèches/school feeding schemes.

Table 3 categorises our findings according to the concepts of the framework developed for this study (Figure 1): social needs, sources of variation, and services.

Table 3. Overview of study findings according to concepts of the framework described in Figure 1.

Social needs	Sources of variation	Services

For peer review only - http://bmjopen.bmj.com/site/about/guidelines.xhtml

**1. Financial planning for nutrition**	- Ability to save, maintain and multiply budget<br>- Ability to trade off present investment for future return<br>- Know-how of eating healthy on a budget (i.e., buying in bulk, home preparation)	- Social grants<br>- Stokvel<br>- Poverty alleviation projects<br>- Skills training<br>- Food aid (vouchers)
**2. Personal income stability**	- Single motherhood/absent fathers<br>- Grant-, kinship-, loan-dependency<br>- Lack of control over nutrition in the household	- Clinic-based service (nutrition advice)<br>- Social grants<br>- Stokvel<br>- Crèches/school feeding scheme
**3. Appropriate and affordable housing**	- Monthly rent payments<br>- Overcrowding, sharing of scarce resources<br>- Household's food budget must cater for all vs individuals' nutritional needs<br>- Poor living conditions (i.e., lack of stable electricity, storage space, refrigeration)	- Social grants<br>- Food aid<br>- Clinic-based service (nutrition advice)<br>- Stokvel
**4. Access to civic services, welfare, and labour market**	- Service eligibility (i.e., possession of identification documents, birth certificate, matric certificate)<br>- School dropouts<br>- Life skills (i.e., internet literacy, CV writing, form completion)<br>- Ability to pay for transportation, printing, internet	- Social grants<br>- Food aid<br>- Formal employment<br>- Skills training
**5. Social support**<br>*5.1 Psychosocial*	- Healthcare and welfare stigma<br>- Low self-esteem and hopelessness<br>- Poor coping (mental health and substance abuse)<br>- Poor partner and family dynamics<br>- Domestic violence	- Clinic-based services (antenatal care, social workers)<br>- Food aid<br>- Social grants
5.2 Social cohesion and connectedness	- Perceived and experienced mistrust, corruption, exclusion<br>- Lack of belonging, isolation<br>- Social connectedness (i.e., group membership)	- Food aid<br>- Poverty alleviation projects<br>- Skills training<br>- Crèches/school feeding scheme
**6. Affordable healthier foods**	- Delayed/periodic access to healthier food<br>- Ability to pay for transport to supermarkets<br>- Awareness of healthier diet (beyond fruits and vegetables i.e., legumes, brown rice)<br>- Influence of local food environment	- Clinic-based service (nutrition advice)<br>- Social grants

<u>Financial planning for nutrition</u>

Participants saw nutrition and unmet social needs for financial planning as deeply intertwined. Mothers' ability to save and/or to maintain and multiply scarce nutrition budgets was perceived to determine the impact of five services on nutrition. First, the degree to which a mother could "stretch" scarce resources including social grants and food vouchers was partly determined by her ability to create and maintain a household budget. A CBO participant emphasised that: *"If we give them food vouchers, we must make sure that they're able to stretch their food. That is why budgeting is important"*. Without financial skills, mothers were perceived to *"spend a long time without cash between paycheques. By the time they reach month end, the food is long finished"* (FGD 2).

For peer review only - http://bmjopen.bmj.com/site/about/guidelines.xhtml

Second, well planned finances allowed some mothers to save for start-up costs, necessary to fund their poverty alleviation projects. Savings allowed mothers to buy seedlings and tools for gardening projects, or ingredients for a baking group. A CBO participant claimed that mothers without financial skills struggle to plan, *"if they've got ZAR 100 [USD 6], instead of buying seeds to invest for the future, they will rather think for today."*

It was also reported that mothers with financial know-how utilized social grants and food vouchers in a manner that was more beneficial for nutrition. This is because financially literate mothers spent more effectively on nutrition, purchased in bulk, looked for discounts, and cut expenses by preparing food at home instead of purchasing ready-made baby foods at high costs. Resulting savings were perceived to be used to diversify diets.

Not all financially literate mothers benefitted from services to the same degree. Participants acknowledged that transforming small amounts of money into savings was harder for those with low and uncertain incomes. Services with long-term return on investments such as skills training workshops, or those that required sustained monetary contribution such as stokvels saw larger variation in mothers' capability to benefit.

Personal income stability

The effect of available services on nutrition was seen to be dependent on if "*one has another form of cash except for the social grant"* (FGD 5). For example, clinic-based nutrition advice was seen valuable but its potential effect diminished when mothers lacked personal income stability, as one CBO participant explained: *"if you are saying to a mother who has just given birth, they must go get fruits, vegetables. They don't have income for that. In fact, they are waiting for the first grant money".* Moreover, without personal income stability, some mothers lived in overcrowded households, where cohabitation compromised mothers' decision-making power over nutrition and restricted their capability to implement any nutrition advice. A CBO participant explained that as a mother who is dependent on others in the household *"You must take whatever is readily available for you. Even if you know that eating maize every day is not good. […] Maybe today you wanted to say 'can you try fish' - you don't have that voice because you're not financially contributing*".

Other service benefits that were contingent on mothers' personal income stability, included those that required sustained financial contribution. One such service was crèches with nutritious meals. Mothers recognized these as valuable for children to be well nourished, however not every mother and child could benefit from them. A participant suggested that as a grant dependent mother *"You can't take them [children] to a crèche because the social grant money has to buy food"* (FGD 4).

Similarly, participation in stokvels required monthly contributions that only mothers with adequate income stability could afford and benefit from. Participants highlighted that a mother without financial stability *"will tell you that they [stokvels] will make her cash short for the rest of the month"* (FGD 6). By contrast, those who were able to benefit from stokvels were seen to be more food secure. This was *"because come January until around March you still have food. And if there a few of you in the house, say about 4 of you – it can easily last until July"* (FGD 4).

Affordable and appropriate housing

Mothers with unmet needs for affordable and appropriate housing were perceived to benefit less from the potential effect of four services on nutrition. First, the degree to which a mother could convert social grants to improved nutrition was dependent on whether she had to pay rent. In the absence of personal income or father's financial support, cost-burdened mothers relied on the Child Support Grant to cover all household expenses including rent. Participants recognized this as a key source of variation in mothers' capability to purchase the foods recommended at the clinic: "*After pay day, one can maybe buy those [recommended] fruits for the first and second week. But how much will*

For peer review only - http://bmjopen.bmj.com/site/about/guidelines.xhtml

one have left from the grant money after paying the rent. These are the reasons we say we are not the same" (FGD 4).

Second, participants explained that while *"we all get the social grant",* mothers' capability to use the grant to enhance nutrition differed when *"there's just 3 of them in a household, while others there are 10 of them in a household"* (FGD 3). Lack of affordable housing inevitably resulted in overcrowding. With many mouths to feed social grants and food aid did not last long. It seemed unrealistic that the Child Support Grant or food aid would be safeguarded only for the mother and child because *"that grant has to stretch for everyone, even for the unborn child"* (CBO participant). Mothers' and children's nutrition needs based on clinic recommendations could not be met. Participants explained that as a mother, *"after giving birth you would still stay with other people, so you won't be able to buy something that accommodates only you and say: I have a child and therefore have to put this type of food aside, that I have to eat"* (FGD 2).

Furthermore, some mothers lived in a household without stable electricity, refrigeration, and inadequate storage space. These hindered mothers' capability to purchase fresh produce because *"if you buy something [fresh produce] at Bara [locally], it won't last you a long time [...] since we don't have electricity, food gets rotten"* (FGD 6). Even the storage of perishable food was a problem. This restricted mothers' participation in stokvels where food was usually purchased in bulk at the end of the year. Participant recognized both the potential benefits of stokvels as well as their own inability to participate*: "some can buy a lot of the grocery that is not so perishable, and they will last for six months. The problem is we live in small houses and have storage issue"* (FGD 6).

<u>Access to civic services, welfare, and the labour market</u>

It was believed that some variation in mothers' capability to benefit from services could be avoided if barriers in access to civic services, welfare, and labour market were removed. First, participants discussed access in terms of time and transportation costs. Located outside of the township, access to employment opportunities and government departments required mothers to spend a significant amount of their time on traveling, and between ZAR 20-100 (USD 1-6) on transportation fees. As a CBO participant explained, *"Let's say the ZAR 350 [USD 22] SASSA [South African Social Security Agency] money is in. I'm hungry now, I know the money is there, but I don't have transport to go get the money. That already affects nutrition because I don't have access".* Similarly valuable yet often inaccessible services were the Department of Social Development's food relief and social work services, the Department of Human Settlements for subsidised housing and the Department of Home Affairs for civic services. The latter was of particular concern since without correct documentation (identification document, birth certificates) mothers and children could not qualify for social grants and food parcels. Furthermore, those without identity documents were also unable to obtain a matric certificate on secondary school completion that further restricted mothers' access to the labour market. This was seen to perpetuate a low-skill/low-income trap for some mothers. Besides constraints in physical access, mothers differed by their level of life skills. This manifested in low levels of internet literacy, not knowing how to fill out necessary documents for government services, where to look for employment opportunities, how to write job applications and CVs. These processes were also not without costs (i.e., printing, internet data), and required some degree of technological skills that further prohibited some low-skill/low-income mothers.

<u>Social support</u>

Social cohesion and connectedness

Social groups were seen as a potential buffer against food insecurity, stress, isolation, and lack of belonging in the community. A FGD participant described how one could benefit from being part of a group: *"at our church we contribute something every month – being it tinned-food or anything else [...] we take the groceries to those needy families we know of. We also sit down as women and talk about*

For peer review only - http://bmjopen.bmj.com/site/about/guidelines.xhtml

the challenges we have" (FGD 3). Mothers who were socially connected were seen more resourceful as information about potential opportunities (including jobs, income generating groups, entrepreneurial advice) were circulated among these social networks. A CBO participant noted that *"some of the resources are in churches, some are in other people just saying, 'I can show you how to write a CV' […] that empowers you to then be able to look for a job better".* Nevertheless, FGD participants reported that lack of trust and feelings of exclusion limited some mothers' capability to access and utilize certain resources. FGD participants saw poverty alleviation projects and food aid as important services for nutrition but of lower value due to their feelings of mistrust. Benefits of these services were seen contingent on having close ties with service providers.

Age was also believed to contribute to variation in mothers' capability to benefit from employment opportunities, and poverty alleviation projects. Both CBO and FGD participants saw older mothers (35 >) being able to benefit less from economic opportunities, while younger FGD participants felt excluded from poverty alleviation projects. A participant illustrated this point by sharing her experience of trying to join a gardening group: *"The granny I asked if I can't just plant even if it is a few onions on the side, said that it was for the elderly. She said I could -because I was still young - go find a job in an office"* (FGD 6). When opportunities were filled or groups were full, FGD participants saw this as the manifestation of "corruption" in the community. FGD participants' observations on this resonated with that of CBO representatives. However, perspectives differed about why this was the case. CBOs emphasized the scarcity of poverty alleviation projects in the community, hence opportunities filled up fast. Further, CBOs' eligibility criteria for beneficiaries may be leaving mothers feeling excluded and believing that CBOs *"have their own people"* (FGD 2).

Psychosocial support

The degree to which mothers benefitted from clinic-based services depended on their perception of the stigma associated with seeking help. Engagement with social workers at clinics was associated with fear of judgement by others in the community. The negative attitude of healthcare workers towards young mothers at antenatal and postnatal visits often left mothers feeling devalued and unaccepted. Low self-esteem was also associated with young women's position in their households where one's acceptance and value were contingent on financial contribution. As such, despite the theoretical advantages of social support, large family networks did not seem to translate into benefit for mothers. Participants described displays of negative attitudes of family members when mothers, especially teenagers, had babies. Additional expenses related to the new-born were often perceived as cumbersome for low-income families. Resulting inadequate financial and childcare support for these young mothers were seen as significant contributors to school dropouts, and a barrier to participate in skills training or income generating activities. Furthermore, those without adequate psychosocial support were more likely to engage in risky coping strategies including substance abuse, gambling, debt and engaging in abusive relationships. The benefit of food vouchers, and social grants for mother's and children's nutritional status were limited in circumstances where these scarce resources were spent on harmful habits or when abusive relationships took away mothers' decision-making power.

<u>Access to affordable healthier foods</u>

Participants confirmed that most mothers in the community were aware and willing to follow clinic-based nutrition advice and wished to spend social grants in a more "suitable" way for nutrition. However, their capabilities to execute their decisions was hindered by lack of finances. FGD participant explained, *"even though we can be told the same advice- our pockets sizes are not the same. I don't have the money to buy the things I was advised to buy"* (FGD 4). Foods recommended at the clinics were perceived to be expensive and being able to afford them was the exception rather than the norm. Access to healthier food was restricted to a few days in a month when mothers received the Child Support Grant, as explained by a FGD participant: *"it's only when you get the social grant money*

For peer review only - http://bmjopen.bmj.com/site/about/guidelines.xhtml

that you can buy that, then you have at least a banana and yoghurt. During the month, you don't have" (FGD 6). Despite fruits and vegetables being available locally at a low cost, FGD participants explained that mothers *"have to go to Woolworths [high-end supermarket], because veggies here at the township are not fresh"* (FGD 1). However, mothers' access to supermarkets depended on their ability to pay for transportation. When the opportunity cost of accessing healthier foods became too high, some mothers had to compromise the quality of food over its quantity. FGD 2 participant explained that *"nutritional food is more expensive. And then its quantity becomes little. When you look at the families there are many of us. […] How long will it last. So, when you buy, you weigh your options. Even with the Rama [margarine]. You buy a cheap one, which does not have the right nutrition."* Mothers with limited access to supermarkets relied on local shops that were described as sources of inexpensive but unhealthy foods. *"They sell kotas [street food]– There's nowhere they sell sandwiches, salads"* (FGD 4).

Discussion

Despite free primary healthcare services and a well-developed social protection system for mothers and children, there are significant inequalities in nutrition outcomes across South Africa. Much attention has been paid to addressing nutrition inequalities through increasing universal health coverage (12,13), and through removing access barriers to services (34,35). Less consideration has been given to what determines the realization of benefits, once services are available and accessible. We developed a framework anchored in ecological and capability approaches and defined social needs as the requisites that can magnify (if unmet) or reduce (if met) variation in the degree to which individuals can benefit from existing services. We applied this framework to identify social needs that determine maternal and child nutrition through engaging pregnant women, mothers, and CBOs in an urban township community.

From a policy perspective, this is relevant, because the extent to which existing services could enhance maternal and child nutrition will be moderated by mothers' capability to benefit from these services. To this end, our study fills several gaps in the literature including what drives persisting nutrition inequalities; and in terms of how we design nutrition policies. Recent commentaries have highlighted the limitations of current micro and macro level approaches in understanding health inequalities (36,37). Academics have called for new narratives that take account of *"the variation within groups as a way to better understand the dynamics of inequality"* (Lundberg 2020, 36). Our social needs approach locates these variations at the meso level of the ecological model and highlights how the meso level forms a link between the macro level and micro level in producing patterns of nutrition inequalities (Figure 1). This is key, because to date, studies have focused on the absolute effect of determinants and services (macro level) on nutrition (micro level). Furthermore, efforts to increase access and utilization of services to improve nutrition in LMICs focused mostly on health sector interventions (34,35). The social needs approach extends this focus to multiple sectors by considering all types of services that are perceived as valuable for mothers to ensure optimal nutrition.

We found that in an urban township, the capability of mothers to benefit from eight valued services to keep themselves and their children well-nourished depended on their ability to meet six social needs. Of these, some needs for example personal income stability, have been identified before. Previous studies have recognized financial constraints as a key barrier for some mothers to access healthier foods in Soweto (38,39). These have frequently been linked to lack of financial support from fathers, and consequent dependency on social grants (38–40). Previous analyses have revolved around the impact of financial constraints on food related perceptions, purchasing and dietary behaviours. The impact of financial barriers on individuals' interactions with available services that they see as valuable for nutrition has been less of a focus. The value of the social needs approach in the present study lies not only in establishing that lack of personal finances limited mothers' nutrition, but in identifying which were those services that - when mothers were deprived of - limit optimal nutrition. In our study, nutrition was compromised when mothers without an additional income

For peer review only - http://bmjopen.bmj.com/site/about/guidelines.xhtml

source (other than social grants) were deprived from the benefits of poverty alleviation projects, crèches with access to nutritious meals, and clinic-based nutrition advice. Low income also limited mothers' ability to attend skills training workshops thereby depriving them from the benefits of economic progress and subsequent improved nutrition.

Through the social needs lens, this study extended previously described associations between suboptimal nutrition and unemployment (38) by identifying reasons that keep mothers unemployed, and what it would take to enhance their capability to benefit from available job opportunities. The government's primary strategy to unemployment includes job creation (41). Notwithstanding the need for more jobs at the macro level, we showed that mothers in this study needed more immediate and short-term support including technological skills and internet literacy, writing CVs, preparing for job interviews, and incidental expenses linked to job applications. Like mothers in this study, Maharaj and Dunn et al (2022) found that young Black African women in a urban township in Durban, South Africa, could not afford to go out and search for employment because they did not have the means to do so (42).

We also showed that the same unmet needs constrained the ability of mothers to benefit from various government services. These included civic services (birth certificates, identity documents) as well as welfare (social grants, food parcels, social housing). With regards to the latter, unmet need for appropriate and affordable housing was seen to be responsible for a large share of the variation in benefit from social grants and food aid. Mothers' ability to direct service benefit towards children's nutrition needs was diminished when social grants and food had to cater for many in overcrowded households and cover all household expenses. Nutrition as recommended at the clinic was also compromised when mothers' needs for stable electricity, refrigeration and storage space in the households were unmet. While the link between nutrition and poor living conditions has been discussed in previous studies of the study area, these emphasised the role of poor sanitation and hygiene in malnutrition (38). Here we showed how government resources (social grants and food aid) may lose their effectiveness if needs for affordable and appropriate housing are unmet.

A social need linked to finances, but less well documented, was financial planning skills. Nutrition was compromised when mothers lacked the financial acumen to manage scarce resources including social grants and food vouchers. Well planned finances on the other hand were seen as a protective factor against running out of resources by the end of the month. Furthermore, financially literate mothers were seen to use social grants and food vouchers in a way that was better for nutrition. We identified that meeting mothers' need for financial planning skills could reduce avoidable variation in benefiting from stokvels, poverty alleviation projects, and skills development trainings.

Lastly, a realm of studies has identified social support as imperative to buffer against stress during and after pregnancy and promote healthier mother and child nutrition (43,44). Social support, however, has not been readily available to South African mothers. Like some of the participants in this study, researchers have associated this unmet need with the absence of fathers, negative provider-patient interaction at clinics (44–47), and stigma attached to being pregnant at a young age (40,48). This study extends the literature on stigma by documenting mothers' perceptions of stigma associated with seeking assistance to cope with financial and nutrition difficulties from social workers and/or informal networks. This is key to the discussion around nutrition inequalities because evidence from high income countries has shown how variation in health benefits might be partially explained by the stigmatizing effect of services (49). Furthermore, we showed how mistrust and feelings of exclusion from available services limited mothers' ability to forge support networks that could help to deal with their issues. Participants in this study found food aid and poverty alleviation projects to be important for nutrition but of lower value because they mistrusted the service provider and felt excluded from

For peer review only - http://bmjopen.bmj.com/site/about/guidelines.xhtml

the projects. Mistrust of services in this way also contributes to variation in mother's capacity to benefit from services and produces nutrition inequities as a consequence.

Strengths and limitation

This is the first qualitative study in South Africa to understand the variation of nutrition outcomes of mothers and children through a social needs approach. A key strength of the study design was engaging multiple stakeholder groups that enhanced both the validity and comprehensiveness of the data. Nevertheless, stakeholders' perspectives were not representative of all South African mothers. Participants represented the experiences of lower-income mothers in a resource constrained urban township. Social needs however may take different forms at different locations (i.e., rural areas). For this study purposes, it was important however to demonstrate that social needs are context-dependent, and researchers and policy makers should challenge the way current services are designed and targeted.

Conclusion

Mothers and pregnant women differ in their abilities to benefit from available healthcare services and social protection services. This has implications for them and for their children's nutritional health. Investigating the variation in mothers' ability to benefit from services might help understand why nutrition inequalities persist despite well-developed policies. A social needs approach can help identify what additional support mothers require to turn available services into nutrition benefits. Multi-sectoral strategies are needed to address social needs and to counterbalance narrow clinic-based nutrition services and high-level population instruments. A social needs approach could complement and reduce the variation of in the effect of existing services that contribute to inequalities in mother and child nutrition outcomes.

Contributorship statement

AE conceptualised the study with input from all co-authors. AE and CD developed the study framework, and AE and NC designed the data collection tools. AE collected and analysed the data. NC guided data analysis and interpretation, with input from all co-authors. AE drafted the manuscript, and all co-authors edited the manuscript and approved the final version.

Competing interests

The authors declare that they have no competing interests.

Funding

This research was funded by the National Institute for Health Research (NIHR) (17\63\154) using UK aid from the UK Government to support global health research (https://www.nihr.ac.uk/). The views expressed in this publication are those of the authors and not necessarily those of the NIHR or the UK Department of Health and Social Care. The funders had no role in study design, data collection and analysis, decision to publish, or preparation of the manuscript. AE, CD, and KJH are supported by the SAMRC/Wits Centre for Health Economics and Decision Science—PRICELESS SA (grant number 23108). AE has received funding from the Faculty of Health Sciences, University of the Witwatersrand, Johannesburg, South Africa.

Data sharing statement

The dataset generated and analysed during the current study is not publicly available due to limitations of ethical approval with regards to participant confidentiality but are available from the corresponding author on reasonable request.

Ethics statement

For peer review only

The study was conducted according to the guidelines laid down in the Declaration of Helsinki and all procedures involving research study participants were approved by University of Witwatersrand Ethics Committee (M210718) and the Research Committee of Johannesburg Health District (GP_202110_002). All participants were informed that participation in the study was voluntary and reassured about the confidentiality of the data collected. All participants gave their informed consent.

Acknowledgements
The authors wish to thank Sandra Ntebe for her contribution to the fieldwork and to the participants for taking part in this study.

References

1. World Health Organization. Global Nutrition Targets 2025 [Internet]. [cited 2022 Mar 23]. (Policy Brief Series). Available from: https://www.who.int/publications/i/item/WHO-NMH-NHD-14.2

2. World Health Organization. Low birth weight, prevalence (%) [Internet]. Global Health Observatory. [cited 2022 Nov 11]. Available from: https://www.who.int/data/gho/data/indicators/indicator-details/GHO/low-birth-weight-prevalence-(-)

3. World Health Organization. Stunting prevalence among children under 5 years of age (% height-for-age <-2 SD) [Internet]. Global Health Observatory. [cited 2022 Nov 11]. Available from: https://www.who.int/data/gho/data/indicators/indicator-details/GHO/gho-jme-country-children-aged-5-years-stunted-(-height-for-age--2-sd)

4. World Health Organization. Overweight prevalence among children under 5 years of age (%) (% weight-for-height >+2 SD) (survey-based estimates) [Internet]. The Global Health Observatory. [cited 2022 Oct 3]. Available from: https://www.who.int/data/gho/data/indicators/indicator-details/GHO/gho-jme-overweight-prevalence

5. World Health Organization. Prevalence of anaemia in women of reproductive age (aged 15-49) (%) [Internet]. Global Health Observatory. [cited 2022 Nov 11]. Available from: https://www.who.int/data/gho/data/indicators/indicator-details/GHO/prevalence-of-anaemia-in-women-of-reproductive-age-(-)

6. Onubi OJ, Marais D, Aucott L, Okonofua F, Poobalan AS. Maternal obesity in Africa: a systematic review and meta-analysis. Journal of Public Health. 2016;38(3):e218–31.

7. Nyati LH, Pettifor JM, Norris SA. The prevalence of malnutrition and growth percentiles for urban South African children. BMC Public Health. 2019 May 2;19(1):492.

8. Prioreschi A, Wrottesley SV, Said-Mohamed R, Nyati L, Newell ML, Norris SA. Understanding how maternal social and biological factors are related to fetal growth in an urban South African cohort. Journal of Developmental Origins of Health and Disease. 2021 Feb;12(1):79–87.

9. English R, Peer N, Honikman S, Tugendhaft A, Hofman KJ. 'First 1000 days' health interventions in low- and middle-income countries: alignment of South African policies with high-quality evidence. Glob Health Action. 2017 Jul 18;10(1):1340396.

10. Child support grant | South African Government [Internet]. [cited 2022 Sep 21]. Available from: https://www.gov.za/services/child-care-social-benefits/child-support-grant

11. Diagnostic/Implementation Evaluation of Nutrition Interventions for Children from Conception to Age 5: Final Report [Internet]. Pretoria, South Africa: Department of Health Department

For peer review only

(DoH), Department of Performance Monitoring and Evaluation (DPME), Department of Social Development (DSD); 2014 [cited 2020 Aug 4]. Available from: https://evaluations.dpme.gov.za/evaluations/441

12. Hasan MM, Magalhaes RJS, Ahmed S, Ahmed S, Biswas T, Fatima Y, et al. Meeting the Global Target in Reproductive, Maternal, Newborn, and Child Health Care Services in Low- and Middle-Income Countries. Global Health: Science and Practice. 2020 Dec 23;8(4):654–65.

13. Requejo J, Diaz T, Park L, Chou D, Choudhury A, Guthold R, et al. Assessing coverage of interventions for reproductive, maternal, newborn, child, and adolescent health and nutrition. BMJ. 2020 Jan 27;368:l6915.

14. Bronfenbrenner U. Towards an experimental ecology of human development. American Psychologist. 1977;32(7):513–31.

15. Bronfenbrenner U. Ecological models of human development. International Encyclopedia of Education. 1994;3(2):1643–7.

16. Mahmudiono T, Segalita C, Rosenkranz RR. Socio-Ecological Model of Correlates of Double Burden of Malnutrition in Developing Countries: A Narrative Review. International Journal of Environmental Research and Public Health. 2019 Jan;16(19):3730.

17. Black RE, Victora CG, Walker SP, Bhutta ZA, Christian P, de Onis M, et al. Maternal and child undernutrition and overweight in low-income and middle-income countries. The Lancet. 2013 Aug 3;382(9890):427–51.

18. Scott V, Schaay N, Schneider H, Sanders D. Addressing social determinants of health in South Africa : the journey continues. South African Health Review. 2017;2017(1):77–87.

19. Black RE, Victora CG, Walker SP, Bhutta ZA, Christian P, Onis M de, et al. Maternal and child undernutrition and overweight in low-income and middle-income countries. The Lancet. 2013 Aug 3;382(9890):427–51.

20. Sen. The Quality of Life. Oxford University Press; 1993. 466 p.

21. Sen A. Inequality Reexamined. New York: Russell Sage Foundation; 1992.

22. Bronfenbrenner U. The ecology of human development: Experiments by nature and design. Cambridge, MA: Harvard University Press; 1979.

23. Ballard R, Hamann C. Income Inequality and Socio-economic Segregation in the City of Johannesburg. In: van Ham M, Tammaru T, Ubarevičienė R, Janssen H, editors. Urban Socio-Economic Segregation and Income Inequality: A Global Perspective [Internet]. Cham: Springer International Publishing; 2021 [cited 2022 Jun 27]. p. 91–109. (The Urban Book Series). Available from: https://doi.org/10.1007/978-3-030-64569-4_5

24. Statistics South Africa. Soweto [Internet]. [cited 2023 Feb 6]. Available from: https://www.statssa.gov.za/?page_id=4286

25. Hall K, Richter L, Mokomane Z, Lake L. South African Child Gauge 2018 [Internet]. Cape Town: Children's Institute, University of Cape Town; [cited 2023 Feb 6]. Available from: http://www.ci.uct.ac.za/ci/child-gauge/2018

For peer review only

26. Cele L, Willen SS, Dhanuka M, Mendenhall E. Ukuphumelela: Flourishing and the pursuit of a good life, and good health, in Soweto, South Africa. SSM - Mental Health. 2021 Dec 1;1:100022.

27. Ware LJ, Kim AW, Prioreschi A, Nyati LH, Taljaard W, Draper CE, et al. Social vulnerability, parity and food insecurity in urban South African young women: the healthy life trajectories initiative (HeLTI) study. J Public Health Pol. 2021 Sep 1;42(3):373–89.

28. Department of Social Development. Children's Services Directory, Gauteng, 2016: A Guide to Provincial Non-Profit Organisations and Government Resources for Vulnerable Children. (2nd ed). Pretoria: USAID; 2016.

29. NGO Advisor Group [Internet]. [cited 2022 Feb 1]. Available from: www.ngoadvisor.co.za

30. Morse JM. The Significance of Saturation. Qual Health Res. 1995 May 1;5(2):147–9.

31. MAXQDA 2020 [Internet]. Berlin, Germany: VERBI Software.; 2019. Available from: https://www.maxqda.com/

32. Vaismoradi M, Turunen H, Bondas T. Content analysis and thematic analysis: Implications for conducting a qualitative descriptive study. Nursing & Health Sciences. 2013;15(3):398–405.

33. Tong A, Sainsbury P, Craig J. Consolidated criteria for reporting qualitative research (COREQ): a 32-item checklist for interviews and focus groups. International Journal for Quality in Health Care. 2007 Dec 1;19(6):349–57.

34. Dahab R, Sakellariou D. Barriers to Accessing Maternal Care in Low Income Countries in Africa: A Systematic Review. Int J Environ Res Public Health. 2020 Jun;17(12):4292.

35. Banke-Thomas OE, Banke-Thomas AO, Ameh CA. Factors influencing utilisation of maternal health services by adolescent mothers in Low-and middle-income countries: a systematic review. BMC Pregnancy and Childbirth. 2017 Feb 16;17(1):65.

36. Lundberg O. Next steps in the development of the social determinants of health approach: the need for a new narrative. Scand J Public Health. 2020 Jul 1;48(5):473–9.

37. Dzau VJ, Laitner MH, Balatbat CA. Has traditional medicine had its day? The need to redefine academic medicine. The Lancet. 2022 Oct 22;400(10361):1481–6.

38. Erzse A, Goldstein S, Norris SA, Watson D, Kehoe SH, Barker M, et al. Double-duty solutions for optimising maternal and child nutrition in urban South Africa: a qualitative study. Public Health Nutrition. 2021 Aug;24(12):3674–84.

39. Besselink D, Brandt H, Klingberg S, Draper CE. Perceptions of healthy food, and perceived facilitators and barriers to buying and consuming healthy food, among female caregivers in Soweto, South Africa. South African Journal of Child Health. 2022 Oct 11;172–7.

40. Drysdale RE, Slemming W, Makusha T, Richter LM. Father involvement, maternal depression and child nutritional outcomes in Soweto, South Africa. Maternal & Child Nutrition. 2021;17(S1):e13177.

41. National Planning Commision. National Development Plan 2030 Our Future-make it work [Internet]. [cited 2023 Jan 7]. Available from:

For peer review only

https://www.gov.za/sites/default/files/gcis_document/201409/ndp-2030-our-future-make-it-workr.pdf

42. Maharaj P, Dunn S. “It’s Not Easy Being a Mom Especially When You Are Unemployed”: Navigating Childcare, a Necessary Social Service in South Africa. Journal of Social Service Research. 2022 Jul 4;48(4):472–84.

43. Lee HY, Song IH, Kawachi I. Maternal and child social support and food availability in relation to child growth in four low- and middle-income countries. Sci Rep. 2022 Apr 8;12(1):5910.

44. Mlotshwa L, Manderson L, Merten S. Personal support and expressions of care for pregnant women in Soweto, South Africa. Global Health Action. 2017 Jan 1;10(1):1363454.

45. Mannava P, Durrant K, Fisher J, Chersich M, Luchters S. Attitudes and behaviours of maternal health care providers in interactions with clients: a systematic review. Globalization and Health. 2015 Aug 15;11(1):36.

46. Silal SP, Penn-Kekana L, Harris B, Birch S, McIntyre D. Exploring inequalities in access to and use of maternal health services in South Africa. BMC Health Services Research. 2012 May 21;12(1):120.

47. Tlebere P, Jackson D, Loveday M, Matizirofa L, Mbombo N, Doherty T, et al. Community-Based Situation Analysis of Maternal and Neonatal Care in South Africa to Explore Factors that Impact Utilization of Maternal Health Services. Journal of Midwifery & Women’s Health. 2007;52(4):342–50.

48. Sewpaul R, Crutzen R, Dukhi N, Sekgala D, Reddy P. A mixed reception: perceptions of pregnant adolescents’ experiences with health care workers in Cape Town, South Africa. Reproductive Health. 2021 Aug 4;18(1):167.

49. Lapham J, Martinson ML. The intersection of welfare stigma, state contexts and health among mothers receiving public assistance benefits. SSM Popul Health. 2022 Jun;18:101117.

For peer review only

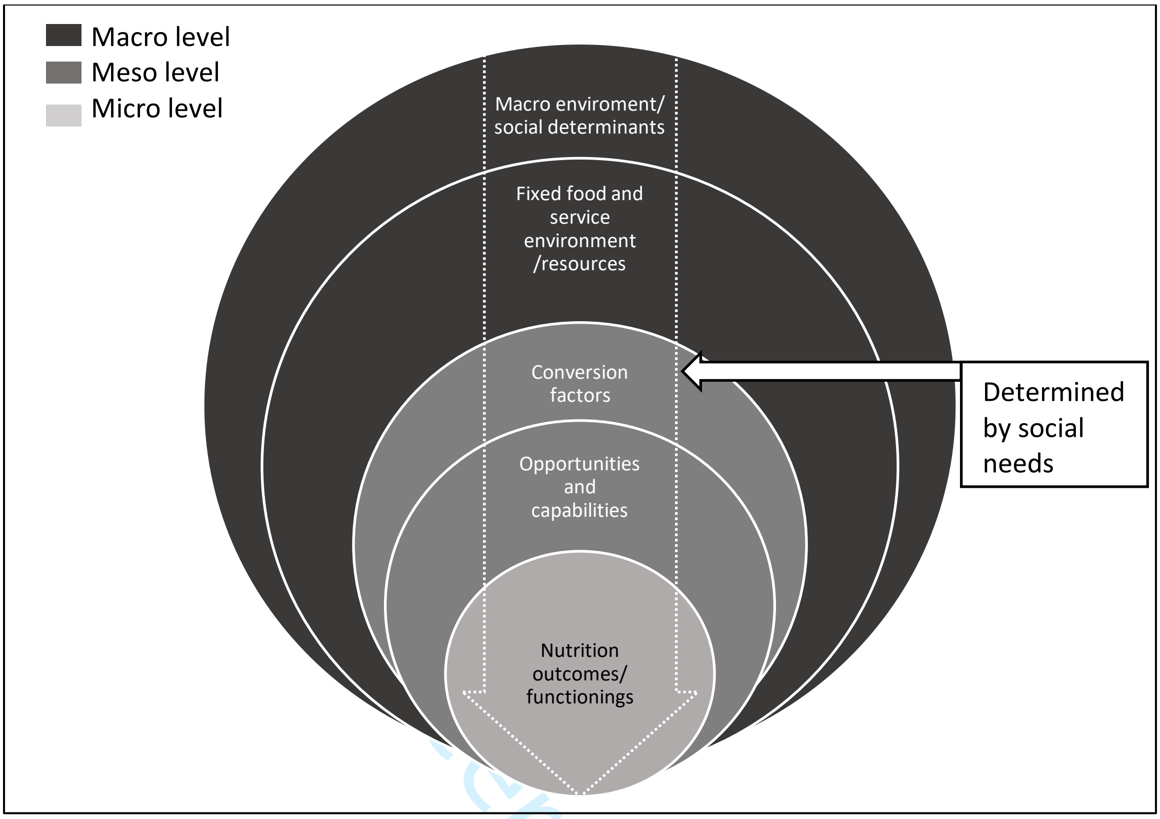

Figure 1. A social needs framework combining the capabilities approach (20) and the ecological model (22)

For peer review only

Appendix A

Focus group compositions

FGD no.	Participants (n)	Age (min-max)	Pregnancy status	Participants (n) per selected community health centres
1	7	22-40	Pregnant	Chiawelo (1), Itireleng (1), Lillian Ngoyi (-), Mofolo (3), Zola (2)
2	8	22-40	Not pregnant	Chiawelo (3), Itireleng (3), Lillian Ngoyi (-), Mofolo (2), Zola (2)
3	7	19-47	Not pregnant	Chiawelo (1), Itireleng (1), Lillian Ngoyi (1), Mofolo (3), Zola (1)
4	8	18-37	Pregnant	Chiawelo (1), Itireleng (3), Lillian Ngoyi (2), Mofolo (1), Zola (1)
5	8	26-49	Not pregnant	Chiawelo (1), Itireleng (2), Lillian Ngoyi (2), Mofolo (1), Zola (2)
6	7	22-48	Not pregnant	Chiawelo (1), Itireleng (3), Lillian Ngoyi (-), Mofolo (1), Zola (2)
7	6	19-42	Pregnant	Chiawelo (1), Itireleng (1), Lillian Ngoyi (2), Mofolo (2), Zola (-)

For peer review only

BMJ Open

A qualitative exploration of the constraints on mothers' and pregnant women's ability to turn available services into nutrition benefits in a low resource urban setting, South Africa

Journal:	*BMJ Open*
Manuscript ID	bmjopen-2023-073716.R1
Article Type:	Original research
Date Submitted by the Author:	25-Sep-2023
Complete List of Authors:	Erzse, Agnes ; University of the Witwatersrand Johannesburg Faculty of Health Sciences, SAMRC/Centre for Health Economics and Decision Science- PRICELESS SA<br>Desmond, Chris; University of the Witwatersrand Johannesburg Faculty of Health Sciences, SAMRC/Centre for Health Economics and Decision Science- PRICELESS SA<br>Hofman, Karen; University of the Witwatersrand Johannesburg Faculty of Health Sciences, SAMRC/Centre for Health Economics and Decision Science- PRICELESS SA<br>Barker, Mary; University of Southampton Faculty of Environmental and Life Sciences, School of Health Sciences; University of Southampton Faculty of Health Sciences, MRC Lifecourse Epidemiology Centre<br>Christofides, Nicola; University of the Witwatersrand Johannesburg Faculty of Health Sciences
**Primary Subject Heading**:	Qualitative research
Secondary Subject Heading:	Public health, Health policy, Health services research
Keywords:	Health Equity, Health policy < HEALTH SERVICES ADMINISTRATION & MANAGEMENT, Community child health < PAEDIATRICS, PUBLIC HEALTH, QUALITATIVE RESEARCH

SCHOLARONE™
Manuscripts

I, the Submitting Author has the right to grant and does grant on behalf of all authors of the Work (as defined in the below author licence), an exclusive licence and/or a non-exclusive licence for contributions from authors who are: i) UK Crown employees; ii) where BMJ has agreed a CC-BY licence shall apply, and/or iii) in accordance with the terms applicable for US Federal Government officers or employees acting as part of their official duties; on a worldwide, perpetual, irrevocable, royalty-free basis to BMJ Publishing Group Ltd ("BMJ") its licensees and where the relevant Journal is co-owned by BMJ to the co-owners of the Journal, to publish the Work in this journal and any other BMJ products and to exploit all rights, as set out in our licence.

The Submitting Author accepts and understands that any supply made under these terms is made by BMJ to the Submitting Author unless you are acting as an employee on behalf of your employer or a postgraduate student of an affiliated institution which is paying any applicable article publishing charge ("APC") for Open Access articles. Where the Submitting Author wishes to make the Work available on an Open Access basis (and intends to pay the relevant APC), the terms of reuse of such Open Access shall be governed by a Creative Commons licence – details of these licences and which Creative Commons licence will apply to this Work are set out in our licence referred to above.

Other than as permitted in any relevant BMJ Author's Self Archiving Policies, I confirm this Work has not been accepted for publication elsewhere, is not being considered for publication elsewhere and does not duplicate material already published. I confirm all authors consent to publication of this Work and authorise the granting of this licence.

For peer review only

Title: A qualitative exploration of the constraints on mothers' and pregnant women's ability to turn available services into nutrition benefits in a low resource urban setting, South Africa

Authors: Agnes Erzse[1*], Chris Desmond[1], Karen Hofman[1], Mary Barker[2,3,4], Nicola Christofides[4]

[1]SAMRC/Wits Centre for Health Economics and Decision Science—PRICELESS SA, School of Public Health, Faculty of Health Sciences, University of Witwatersrand, Johannesburg, South Africa
[2] School of Health Sciences, Faculty of Environmental and Life Sciences, University of Southampton, Southampton, UK
[3]MRC Lifecourse Epidemiology Centre, Faculty of Medicine, University of Southampton, UK
[4]Faculty of Health Sciences, University of the Witwatersrand, Johannesburg, South Africa

Corresponding author: Agnes Erzse, agnes.erzse@wits.ac.za, +27715472158, 27 St. Andrews Road, Parktown, Johannesburg 2193, Republic of South Africa

Key words: maternal and child health, social need, nutrition, inequality, capabilities approach, South Africa

Abstract

Objectives: Despite free primary healthcare services and social protection system for mothers and children, significant nutritional inequalities occur across the globe, including in South Africa. This study aimed to explore what determines mothers' ability to access and turn available services into nutrition benefits.

Design: An exploratory qualitative study was conducted including semi-structured interviews with employees from community-based organizations and focus groups with pregnant women and mothers. Discussions focused on existing services perceived as important to nutrition, differences in mothers' ability to benefit from these services, and the underlying unmet needs contributing to these disparities. Data was analysed thematically using a novel social needs framework developed for this study where social needs are defined as the requisites that can magnify (if unmet) or reduce (if met) variation in the degree to which individuals can benefit from existing services.

Setting: A resource constrained urban township, Soweto in Johannesburg.

Participants: Thirty mothers of infants (<1 year old) and 21 pregnant women attending five primary healthcare facilities participated in seven focus groups, and 18 interviews were conducted with employees from 10 community-based organizations.

Results: Mothers identified social needs related to financial planning, personal income stability, appropriate and affordable housing, access to government services, social support, and affordable healthier foods. The degree to which these needs were met determined mothers' capabilities to benefit from eight services. These were clinic-based services including nutrition advice and social work support, social grants, food aid, community savings groups, poverty alleviation projects, skills training workshops, formal employment opportunities and crèches/school feeding schemes.

Conclusion: Findings demonstrate that while current social protection mechanisms and free health services are necessary, they are not sufficient to address nutrition inequalities. Women's social needs must also be met to ensure that services are accessed and used to improve the nutrition of all mothers and their children.

Strengths and limitations of this study

For peer review only

For peer review only - http://bmjopen.bmj.com/site/about/guidelines.xhtml

- Current approaches to understanding and addressing nutrition inequalities are limited; new methods are needed that consider the variation in individuals' ability to benefit from available services.
- This is the first study in South Africa to understand the variation of nutrition outcomes of mothers and children through a social needs approach.
- This paper reports on the development and application of a novel framework anchored in a an ecological and capabilities approach, to help better understand social needs that limit mothers' ability to turn available services into better nutrition outcomes.
- Perspectives of mothers of young children, pregnant women, and community-based organizations were included, enhancing both the validity and comprehensiveness of the data.
- Participants' views represented the experiences of lower-income mothers in a resource constrained urban township; there is a need to conduct further studies at different locations, where mothers' social needs may take different forms.

Introduction

Poor maternal and child nutrition is one of the biggest preventable causes of ill health and poor human development globally. Despite its commitment to the 2025 global nutrition targets set by the World Health Assembly [1], South Africa is not on track to achieve a 30% reduction in low birthweight and 40% in stunting. Over the past decade, no progress has been made with low birthweight remaining the same; from the benchmark of 14.8% and current prevalence of 15% [2]. Stunting among under five years has risen from 23% in 2012, to 27% of children under five being stunted in 2016 [3]. Moreover, under five childhood overweight and obesity rates are essentially unchanged [4]. Similarly, no progress has been made achieving a 50% reduction in anaemia in women from 28.6% in 2012, with current figures being at 30% [5] and 33% of pregnant women are obese or overweight and 9% have gestational diabetes [6].

There are significant inequalities in nutrition outcomes across and within countries. Disparities are particularly evident in South Africa. Among those at the bottom end of the distribution are mothers and children in lower-income urban townships, have worse nutrition outcomes than the national average. Soweto for example, the largest township in South Africa, has more low birthweight babies (1.6 percentage points), more stunted and obese children under the age of five (7 and 11 percentage points respectively) [7], and more obese mothers (33%) [8].

These disparities exist despite free maternal and child health care; the presence of high impact first 1000 days interventions [9], and social protection mechanisms such as the Child Support Grant (South African Rand [ZAR] 450 or USD 26/month as of 2023) [10]. This suggests that the availability of universal primary care services may not be enough to ensure good nutrition for all mothers and their children. Families may need to access different types and amounts of services and resources such as policies, foods, changes in social norms, and infrastructure, to achieve the same level of optimal nutrition. This variation challenges the way we think about prioritizing and investing in interventions for nutrition. Both globally and in South Africa, the focus has been on increasing the coverage of individual level curative and population level preventative nutrition programmes [11–13]. Less consideration has been given to what makes it possible for individuals to benefit from these existing services.

If nutrition is influenced by variations in needs and opportunities mothers' and pregnant women face, a new theoretical approach that embraces this complexity is necessary. The aim of this paper is to examine these variations through engagement with key stakeholders (mothers, pregnant women, and

For peer review only - http://bmjopen.bmj.com/site/about/guidelines.xhtml

community-based organizations) in Soweto and to operationalize a novel conceptual framework combining ecological and capabilities approaches.

Existing theoretical approaches: missing social needs?

Bronfenbrenner (1977) was one of the earliest theorists to capture the complexities of human development through his ecological model comprised of an arrangement of nested circular systems [14]. His model posits that an individual's development is the result of the dynamic interplay among multiple systems of influence, including the micro- (inner-most circle), meso-, exo-, and macrosystem outer-most circle). A fundamental principle that sets the micro- and mesosystem apart from the exo- and macrosystems is the extent to which these reflect individual experience. Bronfenbrenner envisioned that the micro- and mesosystems refer to the face-to-face interaction of individuals with the immediate settings such as home, neighbourhood, and social networks. By contrast, the exo- and macrosystem consist of social structures (e.g., institutions like government agencies) that regulate individuals' lives at the meso and micro level yet are removed from the individual [15]. Bronfenbrenner's micro, meso, and macro levels have become the foundation for common typologies in the public health literature to describe the drivers of, and strategies to improve, nutrition [16]. Table 1 summarizes some of the most used typologies in the nutrition literature as they relate to the ecological levels.

Table 1. Overview of common typologies used to describe drivers of and interventions for nutrition with examples

Ecological model	Determinants	Streams	Specificity
Micro level	Nutrition needs (i.e., iron deficiency)	Downstream (i.e., iron supplementation)	Nutrition specific
Meso level	Social needs (i.e., transportation, housing)	Mid-stream (i.e., community-based services)	Nutrition sensitive
Macro level	Structural/social determinants of health (i.e., poverty)	Upstream (i.e., social safety nets)	

At the micro level, there are immediate determinants of nutrition (i.e., iron deficiency) which are commonly addressed through medical, nutrition specific actions (i.e., iron supplementation) [17]. Using the metaphor of a stream [18], these actions can be described as downstream. By contrast, upstream actions are located at the macro level and are viewed as nutrition-sensitive [17] as they address the underlying structural and social determinants of health, such as poverty and unemployment. Interventions addressing social determinants of health range from education policies, taxes on unhealthy foods to interventions to improve women's status in society [17], all of which do not involve the individual directly.

Notwithstanding the need for services at the micro and macro levels to improve nutrition, the inequalities described earlier suggest a need to identify meso level strategies that address sources of variation in individuals' capability to benefit from existing services. Sources of variation could include personal (i.e., possession of identity documents) and socio-economic factors (i.e., cost of healthy food), that can lead to avoidable variation in capability to benefit from services if the associated need – referred to as social need – is not met. In this context, social needs are the requisites that can magnify (if unmet) or reduce (if met) the sources of variation individuals face when benefiting existing services. As such, variations in social needs influence the effects of both macro and micro level services on nutrition and may therefore be responsible for consequent disparities in nutrition outcomes.

For peer review only

There is a clear overlap between social needs and "conversion factors", a concept central to Amartya Sen's "capability approach" [19]. The capability approach focuses on the conversion of resources into individual capabilities (real opportunities) to achieve a valuable combination of human functionings, termed "beings" and "doings". From a nutrition point of view, resources can be services and "beings" and "doings" can include being well-nourished. The capabilities approach recognizes that there are personal, social and environmental aspects of the environment (conversion factors) that either expand or restrict one's real opportunities (capabilities) to convert resources into valued functionings [20]. Similarly, social needs play key roles in determining the degree to which mothers can benefit from government and healthcare services for optimal nutrition. The theoretical complementarity between the ecological model and the capabilities approach provides reason to believe that the integration of the two could help better understand what mothers in their communities need so that they are capable of converting available government and healthcare services into better nutrition outcomes.

A social needs framework

The two models combined (Figure 1) allow for mothers' diversity of needs and capabilities to benefit from existing services. Furthermore, it reflects the interaction between ecological levels by demonstrating how such diversity at the meso level determines the effect of an existing service environment on nutrition.

[place of Figure 1]

Figure 1. A social needs framework combining the capabilities approach [19] and the ecological model [21]

The framework groups all services into a combined service environment at the macro level based on the assumption that a "service is a service" for an individual regardless of whether the service targets downstream or upstream determinants. What the model seeks to highlight is that the availability of a service is not an end in itself. The effect of the service (indicated with the arrow on Figure 1) is determined by the conversion factor as it passes through the meso level that determines mothers' real opportunities (capabilities) to benefit from services. This reflects how mothers' experiences of a given service environment and the degree to which they can convert services into nutrition benefits, varies. One might fully benefit from a service by accessing and implementing it, another might partially benefit by accessing it but not being able to use the benefits it offers, and a third might not be able to access or benefit from the service. Where an individual sits on this benefit scale is determined by their social needs and the degree to which these social needs are met. In this sense, the framework identifies conversion factors as the product of social needs.

It is important to note that not all services will see variation in mothers' capability to benefit (conversion factors), and not all variation will be influenced by the same social needs. By applying this framework, the paper aims to explore which services (either macro or micro) see big variation in mothers' conversion factors (capability to benefit) and identify some of the social needs that shape these conversion factors. This framework is particularly relevant in dealing with the nutrition of disadvantaged people or of socioeconomic groups in unfavourable circumstances or conditions because they are likely to have multiple unmet social needs.

One approach to increasing understanding of social needs is to engage those who have direct experience of these needs. These include mothers and pregnant women, as well as community-based organizations (CBOs), defined as any non-profit organization operating at meso levels in community

For peer review only - http://bmjopen.bmj.com/site/about/guidelines.xhtml

settings. The rationale behind engaging CBOs is that they interact and monitor community members and are therefore able to identify their unique challenges.

In a context of available macro level government and micro level nutrition services in Soweto, Johannesburg, this research sought to understand 1) existing services which are perceived as valuable for nutrition but that not all mothers can benefit from equally, 2) the sources of variation in mothers' capability to benefit from those services, and 3) associated social needs that if met could reduce this variation.

Methods

This cross-sectional, exploratory qualitative study was conducted in Soweto between February and October 2022. It involved semi-structured individual interviews (interviews) with employees from CBOs and focus group discussions (FGD) with adult (>18 years) pregnant women and mothers of infants under one year of age attending antenatal and post-natal clinics.

Setting

Soweto is a densely populated working class township which by 2019 contained about 1.8 million people, one third of Johannesburg's population [22]. Inhabitants are almost entirely black South Africans and 80% of adults are unmarried [23]. Forty percent of households are headed by women and one in two children grow up without their father in the home [24]. In some parts of Soweto, individuals endure household and community violence, crime and extreme poverty with 19% of all households not receiving any income [23]. Lower income areas are characterized by unpaved roads, lack of drainage systems, houses with tin roofs, shared drinking water sources and toilet facilities [25,26]. Cohabitation of several families within a four roomed government house is a common feature, with many accommodated in smaller rooms or shacks built at the back of the house [25]. A 2018 survey found that young mothers in Soweto are particularly vulnerable to poor-quality housing, experience lower levels of education and high rates of unemployment (64%) [26]. Compared to nulliparous women, young mothers are also more likely to face food insecurity and have poorer health outcomes, including higher levels of overweight and obesity [26].

Sampling

The selection of interview participants began by developing a contact list of Soweto-based CBOs from an internet search, including two CBO directories [27,28]. CBOs were identified as eligible if the mission/vision statement and overview of services contained explicit language around any of the following: provision of maternal and child health services, social support and nutrition to children, mothers, and pregnant women. CBOs focusing primarily on older persons, sick people (i.e., HIV and AIDs, TB), disabled people, crime prevention and drug abuse were excluded. Out of the fifteen CBOs that were identified and contacted, 10 responded to the invitation to participate, thus comprising our final sample. An invitation letter was sent to CBOs, followed up with a telephone call. Potential participants put forth by the CBOs were approached by the first author and asked if they were willing to participate in the study.

FGD participants included purposively sampled pregnant women attending antenatal care and mothers with infants under one year of age attending postnatal care at five primary health care facilities in Soweto (supplemental file 1). Clinics were purposefully selected to ensure representation from various suburbs, allowing for a more comprehensive collection of perspectives and experiences. Recruitment was undertaken by a local multi-lingual qualitative researcher with experience of working with mothers in Soweto. The researcher approached individuals in communal areas of the health care facilities (e.g., the main corridor, waiting areas) and invited those interested if they resided in Soweto, they were pregnant or had at least one infant under one year of age, and were 18 years of age and above. The sample size for both the interviews and FGDs was determined by data saturation [29].

For peer review only - http://bmjopen.bmj.com/site/about/guidelines.xhtml

Data collection

An interview and a FGD guide were developed separately to assist data collection (see supplemental file 2 and 3). Guides asked participants about their perceptions of the challenges that affected maternal and child nutrition in the community, as well as the available resources, or services that they knew of and might help addressing these challenges. The first author, who had experience in qualitative interviewing and working with communities in Soweto, conducted the interviews in English, either in person at the CBO or via telephone. The FGDs were facilitated by a trained, multi-lingual researcher, familiar with customs and traditions of the community. FGDs participants expressed themselves in English and in their vernacular languages (Xhosa, Zulu, or Sotho). FGDs were organized after the completion of the interviews, and these took place at a research centre in Soweto. All FGD participants completed a sociodemographic questionnaire. A pilot to test the data collection methods was undertaken with participants who met the eligibility criteria. The pilot was conducted in the study setting. . Results from the pilots were included in the analysis. The interviews and FGDs lasted about an hour and all participants were reimbursed for time and transport. Written consent was taken from all participants, and the study protocol was granted ethical approval by the University of Witwatersrand Ethics Committee (M210718) and the Research Committee of Johannesburg Health District (GP_202110_002).

Patient and public involvement

The data collection followed an iterative process, whereby interviews with CBOs informed the development of the FGD guide. Further, early FGDs resulted in adjustments to subsequent ones to enhance the relevance of the questions. A summary of the findings will be presented for validation to participants and patients (attending antenatal and postnatal care).

Data management and analysis

Interviews and FGDs were audio recorded, transcribed, and translated into English where necessary. Transcripts were analysed progressively to determine the point at which saturation was achieved. The first author used a hybrid (deductive and inductive) approach to build the coding structure and coded the interviews and FGD transcripts with MAXQDA 2022 data analysis software [30] using thematic analysis [31]. Data was triangulated from the interviews and FGDs to identify similarities and differences and looked at convergence patterns to validate interpretation. Once inductive and deductive codes were developed, codes were organized into broader categories based on thematic similarities. Lastly, categories were re-organized by three deductive themes anchored in our study objectives and framework (Figure 1): services valuable for nutrition (but which not all mothers could benefit from), sources of variation, and social needs (see supplemental file 4). The co-authors discussed their interpretations of the data. Verbatim quotes from participants have been selected to illustrate how FGD participants and CBOs accounts were linked to themes. Reporting of the findings adheres to COREQ guidelines [32] (see supplemental file 5).

Results

We present the characteristics of participants then discuss the six social needs identified by the participants as they link to multiple sources of variation in mothers' and pregnant women's (hereinafter mothers) capability to benefit from eight different services that both CBOs and mothers identified as valuable for optimal maternal and child nutrition (hereinafter nutrition). Where results are similar for CBOs and mothers, these are presented without participant specification. When results differ, these are noted and discussed.

Characteristics of CBO participants

We interviewed 18 employees representing 10 CBOs that serviced clients in 11 of 40 Soweto suburbs. Eight CBOs aimed to directly improve the well-being of orphans and vulnerable children and

For peer review only - http://bmjopen.bmj.com/site/about/guidelines.xhtml

adolescents (0-18 years of age), and two CBOs aimed to directly improve the well-being of women, including mothers and pregnant women. On average, there were 2 participants per CBO. All but one participant were women, and they represented Founders and Directors (n=4), managers responsible for directing programs and employees (n=2), social workers (n=11) and a volunteer (n=1) who worked directly with beneficiaries. Thirteen of the participants indicated that they had been employed at their respective CBOs for over 4 years.

Characteristics of FGD participants

Fifty-one women participated in 7 FGDs (see group compositions in supplemental file 1). Twenty-one (41%) were pregnant (12 of them for the first time) and of the 39 non-first-time mothers (71%) had at least 2 children. Twenty-nine (56%) participants were single while 17 (41%) were partnered or married. All but two participants were unemployed, and the majority relied on social grants (64%) as their main source of income. Thirty-five participants (68%) reported their household income to be less than ZAR 3000 (USD 187) per month, 77% being less than ZAR 1000 (USD 57.94). Mothers' living conditions differed. Some lived in shacks/informal dwellings (31%), others in owner-occupied houses (bond house) (21%), government subsidised houses (21%), or single rooms built in the backyard of other households (19%). Thirty-two mothers (62%) had stable electricity, 38 (74%) had a fridge, and 10 (19%) had daily access to internet. On average there were 5 people living in their houses (range 2-11). Less than half (45%) reported being members of community groups, and 10 (43%) of those who did were part of *stokvels* (community money savings groups). Details of FGD participant are provided in Table 2.

Table 2. Sociodemographic characteristics of FGD participants

**Age (years)**		
Minimum-maximum; Mean [SD]	18-49	31 [8]
**Pregnancy status**	**N**	**%**
Pregnant	21	41.1
Non-pregnant	30	58.8
**Number of babies/children cared for**		
0 - first time mother	12	23.5
1	11	21.5
2-4	25	49.0
5 and above	3	5.8
**Marital status**		
Single never married	29	56.8
Married	4	7.8
Partnered	17	33.3
Separated	1	1.9
**Highest level of schooling**		
Primary school	2	3.9
Some high school	20	39.2
Completed high school	25	49.0
Diploma/higher diploma	4	7.8
**Household income**		
ZAR 1000 or less	27	52.9
ZAR 1001- 3000	8	15.6
ZAR 3001-R5000	10	19.6

For peer review only - http://bmjopen.bmj.com/site/about/guidelines.xhtml

ZAR 5001-R10000	3	1.9
ZAR 10001-20000	3	5.8
**Source of income**		
Government grants	33	64.7
Formal employment	2	3.9
Informal employment	2	3.9
Others* in the household	8	15.6
**Type of house**		
Bond house	11	21.5
Government subsidised houses	11	21.5
Shack or informal dwelling	16	31.3
Single outside room	10	19.6
Other	2	3.9
**Number of people in the house**		
Minimum-maximum; Mean [SD]	1-11	5 [2]
**Household assets**		
Stable electricity	32	62.7
Radio	33	64.7
A fridge	38	74.5
Television	38	74.5
Cell phone	33	64.7
Daily access to internet	10	19.6
Car	4	7.8
Clothing sufficient to keep mothers and children warm	38	74.5
**Membership**		
Stokvel	10	19.6
Community garden group	2	3.9
Mothers support group	2	3.9
Women's association	2	3.9
Community service (school development committee)	1	1.9
Religious groups	3	5.8
Any	23	45.0
*Infants'/children's father, grandparents, other adult cohabitants.		

Social needs of mothers

The analysis identified six social needs: financial planning; personal income stability; appropriate and affordable housing; access to civic services, welfare, and the labour market; social support; and affordable healthier foods. The following section reports on each of these needs without implying a priority rank order, as they link to multiple sources of variation that enabled or disabled mothers from benefitting from eight different services. These services were recognized by CBOs and mothers as valuable for nutrition but there was variation in mothers' capability to benefit from them. These included clinic-based services (nutrition advice, social workers); social grants (Child Support Grant, Social Distress Relief); food aid including parcels and vouchers (distributed by the Department of Social Development, churches, CBOs, crèches/schools and clinics); stokvels; poverty alleviation projects (sewing, baking groups and food gardens at schools, and CBOs); skills training workshops; formal employment opportunities including learnerships; and crèches/school feeding schemes.

For peer review only - http://bmjopen.bmj.com/site/about/guidelines.xhtml

Table 3 categorises our findings according to the concepts of the framework developed for this study (Figure 1): social needs, sources of variation, and services.

Table 3. Overview of study findings according to concepts of the framework described in Figure 1.

Social needs	Sources of variation	Services
**Financial planning for nutrition**	- Ability to save, maintain and multiply budget<br>- Ability to trade off present investment for future return<br>- Know-how of eating healthy on a budget (i.e., buying in bulk, home preparation)	- Social grants<br>- Stokvel<br>- Poverty alleviation projects<br>- Skills training<br>- Food aid (vouchers)
**Personal income stability**	- Single motherhood/absent fathers<br>- Grant-, kinship-, loan-dependency<br>- Lack of control over nutrition in the household	- Clinic-based service (nutrition advice)<br>- Social grants<br>- Stokvel<br>- Crèches/school feeding scheme
**Appropriate and affordable housing**	- Monthly rent payments<br>- Overcrowding, sharing of scarce resources<br>- Household's food budget must cater for all vs individuals' nutritional needs<br>- Poor living conditions (i.e., lack of stable electricity, storage space, refrigeration)	- Social grants<br>- Food aid<br>- Clinic-based service (nutrition advice)<br>- Stokvel
**Access to civic services, welfare, and labour market**	- Service eligibility (i.e., possession of identification documents, birth certificate, matric certificate)<br>- School dropouts<br>- Life skills (i.e., internet literacy, CV writing, form completion)<br>- Ability to pay for transportation, printing, internet	- Social grants<br>- Food aid<br>- Formal employment<br>- Skills training
**Social support**<br>*Psychosocial*	- Healthcare and welfare stigma<br>- Low self-esteem and hopelessness<br>- Poor coping (mental health and substance abuse)<br>- Poor partner and family dynamics<br>- Domestic violence	- Clinic-based services (antenatal care, social workers)<br>- Food aid<br>- Social grants
Social cohesion and connectedness	- Perceived and experienced mistrust, corruption, exclusion<br>- Lack of belonging, isolation<br>- Social connectedness (i.e., group membership)	- Food aid<br>- Poverty alleviation projects<br>- Skills training<br>- Crèches/school feeding scheme
**Affordable healthier foods**	- Delayed/periodic access to healthier food<br>- Ability to pay for transport to supermarkets<br>- Awareness of healthier diet (beyond fruits and vegetables i.e., legumes, brown rice)<br>- Influence of local food environment	- Clinic-based service (nutrition advice)<br>- Social grants

<u>Financial planning for nutrition</u>

Participants saw nutrition and unmet social needs for financial planning as deeply intertwined. Mothers' ability to save and/or to maintain and multiply scarce nutrition budgets was perceived to determine the impact of five services on nutrition. First, the degree to which a mother could "stretch"

For peer review only - http://bmjopen.bmj.com/site/about/guidelines.xhtml

scarce resources including social grants and food vouchers was partly determined by her ability to create and maintain a household budget. A CBO participant emphasised that: *"If we give them food vouchers, we must make sure that they're able to stretch their food. That is why budgeting is important" (CBO5, women, Founder and Director).* Without financial skills, mothers were perceived to *"spend a long time without cash between paycheques. By the time they reach month end, the food is long finished" (mother of young infant,* FGD 2*).*

Second, well planned finances allowed some mothers to save for start-up costs, necessary to fund their poverty alleviation projects. Savings allowed mothers to buy seedlings and tools for gardening projects, or ingredients for a baking group. A CBO participant claimed that mothers without financial skills struggle to plan, *"if they've got ZAR 100 [USD 6], instead of buying seeds to invest for the future, they will rather think for today" (CBO8, women, Project manager).*

It was also reported that mothers with financial know-how utilized social grants and food vouchers in a manner that was more beneficial for nutrition. This is because financially literate mothers spent more effectively on nutrition, purchased in bulk, looked for discounts, and cut expenses by preparing food at home instead of purchasing ready-made baby foods at high costs. Resulting savings were perceived to be used to diversify diets.

Not all financially literate mothers benefitted from services to the same degree. Participants acknowledged that transforming small amounts of money into savings was harder for those with low and uncertain incomes. Services with long-term return on investments such as skills training workshops, or those that required sustained monetary contribution such as stokvels saw larger variation in mothers' capability to benefit.

<u>Personal income stability</u>

The effect of available services on nutrition was seen to be dependent on if "*one has another form of cash except for the social grant" (mother of young infant, FGD 5).* For example, clinic-based nutrition advice was seen valuable but its potential effect diminished when mothers lacked personal income stability, as one CBO participant explained: *"if you are saying to a mother who has just given birth, they must go get fruits, vegetables. They don't have income for that. In fact, they are waiting for the first grant money" (CBO6, women, Project manager).* Moreover, without personal income stability, some mothers lived in overcrowded households, where cohabitation compromised mothers' decision-making power over nutrition and restricted their capability to implement any nutrition advice. A CBO participant explained that as a mother who is dependent on others in the household *"You must take whatever is readily available for you. Even if you know that eating maize every day is not good. […] Maybe today you wanted to say 'can you try fish' - you don't have that voice because you're not financially contributing" (CBO1, women, Service provider).*

Other service benefits that were contingent on mothers' personal income stability, included those that required sustained financial contribution. One such service was crèches with nutritious meals. Mothers recognized these as valuable for children to be well nourished, however not every mother and child could benefit from them. A participant suggested that as a grant dependent mother *"You can't take them [children] to a crèche because the social grant money has to buy food" (pregnant woman, FGD 4).*

Similarly, participation in stokvels required monthly contributions that only mothers with adequate income stability could afford and benefit from. Participants highlighted that a mother without financial stability *"will tell you that they [stokvels] will make her cash short for the rest of the month" (mother of young infant, FGD 6).* By contrast, those who were able to benefit from stokvels were seen to be more food secure. This was *"because come January until around March you still have food. And if there a few of you in the house, say about 4 of you – it can easily last until July" (pregnant woman, FGD 4).*

For peer review only - http://bmjopen.bmj.com/site/about/guidelines.xhtml

Affordable and appropriate housing

Mothers with unmet needs for affordable and appropriate housing were perceived to benefit less from the potential effect of four services on nutrition. First, the degree to which a mother could convert social grants to improved nutrition was dependent on whether she had to pay rent. In the absence of personal income or father's financial support, cost-burdened mothers relied on the Child Support Grant to cover all household expenses including rent. Participants recognized this as a key source of variation in mothers' capability to purchase the foods recommended at the clinic: "*After pay day, one can maybe buy those [recommended] fruits for the first and second week. But how much will one have left from the grant money after paying the rent. These are the reasons we say we are not the same" (pregnant woman, FGD 4).*

Second, participants explained that while "*we all get the social grant",* mothers' capability to use the grant to enhance nutrition differed when *"there's just 3 of them in a household, while others there are 10 of them in a household" (mother of young infant, FGD 3)*. Lack of affordable housing inevitably resulted in overcrowding. With many mouths to feed social grants and food aid did not last long. It seemed unrealistic that the Child Support Grant or food aid would be safeguarded only for the mother and child because *"that grant has to stretch for everyone, even for the unborn child"* (CBO participant). Mothers' and children's nutrition needs based on clinic recommendations could not be met. Participants explained that as a mother, *"after giving birth you would still stay with other people, so you won't be able to buy something that accommodates only you and say: I have a child and therefore have to put this type of food aside, that I have to eat" (mother of young infant, FGD 2)*.

Furthermore, some mothers lived in a household without stable electricity, refrigeration, and inadequate storage space. These hindered mothers' capability to purchase fresh produce because *"if you buy something [fresh produce] at Bara [locally], it won't last you a long time […] since we don't have electricity, food gets rotten" (mother of young infant, FGD 6)*. Even the storage of perishable food was a problem. This restricted mothers' participation in stokvels where food was usually purchased in bulk at the end of the year. Participants recognized both the potential benefits of stokvels as well as their own inability to participate*: "some can buy a lot of the grocery that is not so perishable, and they will last for six months. The problem is we live in small houses and have storage issue" (mother of young infant, FGD 6)*.

Access to civic services, welfare, and the labour market

It was believed that some variation in mothers' capability to benefit from services could be avoided if barriers in access to civic services, welfare, and labour market were removed. First, participants discussed access in terms of time and transportation costs. Located outside of the township, access to employment opportunities and government departments required mothers to spend a significant amount of their time on traveling, and between ZAR 20-100 (USD 1-6) on transportation fees. As a CBO participant explained, *"Let's say the ZAR 350 [USD 22] SASSA [South African Social Security Agency] money is in. I'm hungry now, I know the money is there, but I don't have transport to go get the money. That already affects nutrition because I don't have access" (CBO1, women, Service provider).* Similarly valuable yet often inaccessible services were the Department of Social Development's food relief and social work services, the Department of Human Settlements for subsidised housing and the Department of Home Affairs for civic services. The latter was of particular concern since without correct documentation (identification document, birth certificates) mothers and children could not qualify for social grants and food parcels. Furthermore, those without identity documents were also unable to obtain a matric certificate on secondary school completion that further restricted mothers' access to the labour market. This was seen to perpetuate a low-skill/low-income trap for some mothers. Besides constraints in physical access, mothers differed by their level of life skills. This manifested in low levels of internet literacy, not knowing how to fill out necessary documents for government services, where to look for employment opportunities, how to write job applications and CVs. These processes were also not without costs (i.e., printing, internet data), and

For peer review only - http://bmjopen.bmj.com/site/about/guidelines.xhtml

required some degree of technological skills that further prohibited some low-skill/low-income mothers.

Social support

Social cohesion and connectedness

Social groups were seen as a potential buffer against food insecurity, stress, isolation, and lack of belonging in the community. A FGD participant described how one could benefit from being part of a group: *"at our church we contribute something every month – being it tinned-food or anything else […] we take the groceries to those needy families we know of. We also sit down as women and talk about the challenges we have" (mother of young infant, FGD 3).* Mothers who were socially connected were seen more resourceful as information about potential opportunities (including jobs, income generating groups, entrepreneurial advice) were circulated among these social networks. A CBO participant noted that *"some of the resources are in churches, some are in other people just saying, 'I can show you how to write a CV' […] that empowers you to then be able to look for a job better" (CBO10, women, social worker).* Nevertheless, FGD participants reported that lack of trust and feelings of exclusion limited some mothers' capability to access and utilize certain resources. FGD participants saw poverty alleviation projects and food aid as important services for nutrition but of lower value due to their feelings of mistrust. Benefits of these services were seen contingent on having close ties with service providers.

Age was also believed to contribute to variation in mothers' capability to benefit from employment opportunities, and poverty alleviation projects. Both CBO and FGD participants saw older mothers (35 >) being able to benefit less from economic opportunities, while younger FGD participants felt excluded from poverty alleviation projects. A participant illustrated this point by sharing her experience of trying to join a gardening group: *"The granny I asked if I can't just plant even if it is a few onions on the side, said that it was for the elderly. She said I could -because I was still young - go find a job in an office" (mother of young infant, FGD 6)*. When opportunities were filled or groups were full, FGD participants saw this as the manifestation of "corruption" in the community. FGD participants' observations on this resonated with that of CBO representatives. However, perspectives differed about why this was the case. CBOs emphasized the scarcity of poverty alleviation projects in the community, hence opportunities filled up fast. Further, CBOs' eligibility criteria for beneficiaries may be leaving mothers feeling excluded and believing that CBOs *"have their own people" (mother of young infant, FGD 2).*

Psychosocial support

The degree to which mothers benefitted from clinic-based services depended on their perception of the stigma associated with seeking help. Engagement with social workers at clinics was associated with fear of judgement by others in the community. The negative attitude of healthcare workers towards young mothers at antenatal and postnatal visits often left mothers feeling devalued and unaccepted. Low self-esteem was also associated with young women's position in their households where one's acceptance and value were contingent on financial contribution. As such, despite the theoretical advantages of social support, large family networks did not seem to translate into benefit for mothers. Participants described displays of negative attitudes of family members when mothers, especially teenagers, had babies. Additional expenses related to the new-born were often perceived as cumbersome for low-income families. Resulting inadequate financial and childcare support for these young mothers were seen as significant contributors to school dropouts, and a barrier to participate in skills training or income generating activities. Furthermore, those without adequate psychosocial support were more likely to engage in risky coping strategies including substance abuse, gambling, debt and engaging in abusive relationships. The benefit of food vouchers, and social grants for mother's and children's nutritional status were limited in circumstances where these scarce

For peer review only - http://bmjopen.bmj.com/site/about/guidelines.xhtml

resources were spent on harmful habits or when abusive relationships took away mothers' decision-making power.

Access to affordable healthier foods

Participants confirmed that most mothers in the community were aware and willing to follow clinic-based nutrition advice and wished to spend social grants in a more "suitable" way for nutrition. However, their capabilities to execute their decisions was hindered by lack of finances. FGD participant explained, *"even though we can be told the same advice- our pockets sizes are not the same. I don't have the money to buy the things I was advised to buy" (pregnant woman, FGD 4).* Foods recommended at the clinics were perceived to be expensive and being able to afford them was the exception rather than the norm. Access to healthier food was restricted to a few days in a month when mothers received the Child Support Grant, as explained by a FGD participant: *"it's only when you get the social grant money that you can buy that, then you have at least a banana and yoghurt. During the month, you don't have" (mother of young infant, FGD 6).* Despite fruits and vegetables being available locally at a low cost, FGD participants explained that mothers "*have to go to Woolworths [high-end supermarket], because veggies here at the township are not fresh" (pregnant woman, FGD 1).* However, mothers' access to supermarkets depended on their ability to pay for transportation. When the opportunity cost of accessing healthier foods became too high, some mothers had to compromise the quality of food over its quantity. FGD 2 participant explained that *"nutritional food is more expensive. And then its quantity becomes little. When you look at the families there are many of us. […] How long will it last. So, when you buy, you weigh your options. Even with the Rama [margarine]. You buy a cheap one, which does not have the right nutrition" (mother of young infant).* Mothers with limited access to supermarkets relied on local shops that were described as sources of inexpensive but unhealthy foods. "*They sell kotas [street food]– There's nowhere they sell sandwiches, salads" (pregnant woman, FGD 4)*.

Discussion

Despite free primary healthcare services and a well-developed social protection system for mothers and children, there are significant inequalities in nutrition outcomes across South Africa. Much attention has been paid to addressing nutrition inequalities through increasing universal health coverage [33,34], and through removing access barriers to services [35,36]. Less consideration has been given to what determines the realization of benefits, once services are available and accessible. We developed a framework anchored in ecological and capability approaches and defined social needs as the requisites that can magnify (if unmet) or reduce (if met) variation in the degree to which individuals can benefit from existing services. We applied this framework to identify social needs that determine maternal and child nutrition through engaging pregnant women, mothers, and CBOs in an urban township community.

From a policy perspective, this is relevant, because the extent to which existing services could enhance maternal and child nutrition will be moderated by mothers' capability to benefit from these services. To this end, our study fills several gaps in the literature including what drives persisting nutrition inequalities; and in terms of how we design nutrition policies. Recent commentaries have highlighted the limitations of current micro and macro level approaches in understanding health inequalities [37,38]. Academics have called for new narratives that take account of *"the variation within groups as a way to better understand the dynamics of inequality"* (Lundberg 2020, 36). Our social needs approach locates these variations at the meso level of the ecological model and highlights how the meso level forms a link between the macro level and micro level in producing patterns of nutrition inequalities (Figure 1). This is key, because to date, studies have focused on the absolute effect of determinants and services (macro level) on nutrition (micro level). Furthermore, efforts to increase access and utilization of services to improve nutrition in LMICs focused mostly on health sector interventions [39,40]. The social needs approach extends this focus to multiple sectors by considering all types of services that are perceived as valuable for mothers to ensure optimal nutrition.

For peer review only - http://bmjopen.bmj.com/site/about/guidelines.xhtml

We found that in an urban township, the capability of mothers to benefit from eight valued services to keep themselves and their children well-nourished depended on their ability to meet six social needs. Of these, some needs for example personal income stability, have been identified before. Previous studies have recognized financial constraints as a key barrier for some mothers to access healthier foods in Soweto [41,42]. These have frequently been linked to lack of financial support from fathers, and consequent dependency on social grants [41–43]. Previous analyses have revolved around the impact of financial constraints on food related perceptions, purchasing and dietary behaviours. The impact of financial barriers on individuals' interactions with available services that they see as valuable for nutrition has been less of a focus. The value of the social needs approach in the present study lies not only in establishing that lack of personal finances limited mothers' nutrition, but in identifying which were those services that - when mothers were deprived of - limit optimal nutrition. In our study, nutrition was compromised when mothers without an additional income source (other than social grants) were deprived from the benefits of poverty alleviation projects, crèches with access to nutritious meals, and clinic-based nutrition advice. Low income also limited mothers' ability to attend skills training workshops thereby depriving them from the benefits of economic progress and subsequent improved nutrition.

Through the social needs lens, this study extended previously described associations between suboptimal nutrition and unemployment [41] by identifying reasons that keep mothers unemployed, and what it would take to enhance their capability to benefit from available job opportunities. The government's primary strategy to unemployment includes job creation [44]. Notwithstanding the need for more jobs at the macro level, we showed that mothers in this study needed more immediate and short-term support including technological skills and internet literacy, writing CVs, preparing for job interviews, and incidental expenses linked to job applications. Like mothers in this study, Maharaj and Dunn et al (2022) found that young Black African women in a urban township in Durban, South Africa, could not afford to go out and search for employment because they did not have the means to do so [45].

We also showed that the same unmet needs constrained the ability of mothers to benefit from various government services. These included civic services (birth certificates, identity documents) as well as welfare (social grants, food parcels, social housing). With regards to the latter, unmet need for appropriate and affordable housing was seen to be responsible for a large share of the variation in benefit from social grants and food aid. Mothers' ability to direct service benefit towards children's nutrition needs was diminished when social grants and food had to cater for many in overcrowded households and cover all household expenses. Nutrition as recommended at the clinic was also compromised when mothers' needs for stable electricity, refrigeration and storage space in the households were unmet. While the link between nutrition and poor living conditions has been discussed in previous studies of the study area, these emphasised the role of poor sanitation and hygiene in malnutrition [41]. Here we showed how government resources (social grants and food aid) may lose their effectiveness if needs for affordable and appropriate housing are unmet.

A social need linked to finances, but less well documented, was financial planning skills. Nutrition was compromised when mothers lacked the financial acumen to manage scarce resources including social grants and food vouchers. Well planned finances on the other hand were seen as a protective factor against running out of resources by the end of the month. Furthermore, financially literate mothers were seen to use social grants and food vouchers in a way that was better for nutrition. We identified that meeting mothers' need for financial planning skills could reduce avoidable variation in benefiting from stokvels, poverty alleviation projects, and skills development trainings.

For peer review only - http://bmjopen.bmj.com/site/about/guidelines.xhtml

Lastly, a realm of studies has identified social support as imperative to buffer against stress during and after pregnancy and promote healthier mother and child nutrition [46,47]. Social support, however, has not been readily available to South African mothers. Like some of the participants in this study, researchers have associated this unmet need with the absence of fathers, negative provider-patient interaction at clinics [47–50], and stigma attached to being pregnant at a young age [43,51]. This study extends the literature on stigma by documenting mothers' perceptions of stigma associated with seeking assistance to cope with financial and nutrition difficulties from social workers and/or informal networks. This is key to the discussion around nutrition inequalities because evidence from high income countries has shown how variation in health benefits might be partially explained by the stigmatizing effect of services [52]. Furthermore, we showed how mistrust and feelings of exclusion from available services limited mothers' ability to forge support networks that could help to deal with their issues. Participants in this study found food aid and poverty alleviation projects to be important for nutrition but of lower value because they mistrusted the service provider and felt excluded from the projects. Mistrust of services in this way also contributes to variation in mother's capacity to benefit from services and produces nutrition inequities as a consequence.

Strengths and limitation

This is the first qualitative study in South Africa to understand the variation of nutrition outcomes of mothers and children through a social needs approach. A key strength of the study design was engaging multiple stakeholder groups that enhanced both the validity and comprehensiveness of the data. Nevertheless, stakeholders' perspectives were not representative of all South African mothers. Participants represented the experiences of lower-income mothers in a resource constrained urban township. Social needs however may take different forms at different locations (i.e., rural areas). For this study purposes, it was important however to demonstrate that social needs are context-dependent, and researchers and policy makers should challenge the way current services are designed and targeted.

Conclusion

Mothers and pregnant women differ in their abilities to benefit from available healthcare services and social protection services. This has implications for them and for their children's nutritional health. Investigating the variation in mothers' ability to benefit from services might help understand why nutrition inequalities persist despite well-developed policies. A social needs approach can help identify what additional support mothers require to turn available services into nutrition benefits. Multi-sectoral strategies are needed to address social needs and to counterbalance narrow clinic-based nutrition services and high-level population instruments. A social needs approach could complement and reduce the variation in the effect of existing services that contribute to inequalities in mother and child nutrition outcomes.

Data availability statement

The dataset generated and analysed during the study is not publicly available due to limitations of ethical approval regarding participant confidentiality.

Ethics statements

Patient consent for publication

Not applicable.

Ethics approval

The study was conducted according to the principles of the Declaration of Helsinki, and all procedures involving research study participants were approved by the University of the Witwatersrand Human Research Ethics Committee (M210718) and the Research Committee of Johannesburg Health District (GP_202110_002). All participants were informed that participation in the study was voluntary and

For peer review only

For peer review only - http://bmjopen.bmj.com/site/about/guidelines.xhtml

reassured about the confidentiality of the data collected. Written informed consent was gathered from each participant.

Acknowledgements
The authors wish to thank Sandra Ntebe for her contribution to the fieldwork and to the participants for taking part in this study.

Author contributions
AE conceptualised the study with input from CD, KH, MB, NC. AE and CD developed the study framework, and AE and NC designed the data collection tools. AE collected and analysed the data. NC guided data analysis and interpretation, with input from all co-authors. AE drafted the manuscript, and all co-authors edited the manuscript and approved the final version.

Funding
This research was funded by the National Institute for Health Research (NIHR) (17\63\154) using UK aid from the UK Government to support global health research (https://www.nihr.ac.uk/). The views expressed in this publication are those of the authors and not necessarily those of the NIHR or the UK Department of Health and Social Care. The funders had no role in study design, data collection and analysis, decision to publish, or preparation of the manuscript. AE, CD, and KJH are supported by the SAMRC/Wits Centre for Health Economics and Decision Science—PRICELESS SA (grant number 23108). AE has received funding from the Faculty of Health Sciences, University of the Witwatersrand, Johannesburg, South Africa.

Declaration
None.

Conflicts of interest
The authors declare that they have no competing interests.

References
1. World Health Organization. Global Nutrition Targets 2025. 2014, Available: https://www.who.int/publications/i/item/WHO-NMH-NHD-14.2

2. World Health Organization. Low birth weight, prevalence (%). In: Global Health Observatory [online]. 2022, Available: https://www.who.int/data/gho/data/indicators/indicator-details/GHO/low-birth-weight-prevalence-(-)

3. World Health Organization. Stunting prevalence among children under 5 years of age (% height-for-age <-2 SD). In: Global Health Observatory [online]. 2022, Available: https://www.who.int/data/gho/data/indicators/indicator-details/GHO/gho-jme-country-children-aged-5-years-stunted-(-height-for-age--2-sd)

4. World Health Organization. Overweight prevalence among children under 5 years of age (%) (% weight-for-height >+2 SD) (survey-based estimates). In: The Global Health Observatory [online]. 2022, Available: https://www.who.int/data/gho/data/indicators/indicator-details/GHO/gho-jme-overweight-prevalence

5. World Health Organization. Prevalence of anaemia in women of reproductive age (aged 15-49) (%). In: Global Health Observatory [online]. 2022, Available: https://www.who.int/data/gho/data/indicators/indicator-details/GHO/prevalence-of-anaemia-in-women-of-reproductive-age-(-)

For peer review only

6. Onubi OJ, Marais D, Aucott L, *et al.* Maternal obesity in Africa: a systematic review and meta-analysis. Journal of Public Health. 2016;38: e218–e231. 10.1093/pubmed/fdv138. doi: 10.1093/pubmed/fdv138

7. Nyati LH, Pettifor JM, Norris SA. The prevalence of malnutrition and growth percentiles for urban South African children. BMC Public Health. 2019;19: 492. doi:10.1186/s12889-019-6794-1

8. Prioreschi A, Wrottesley SV, Said-Mohamed R, *et al.* Understanding how maternal social and biological factors are related to fetal growth in an urban South African cohort. Journal of Developmental Origins of Health and Disease. 2021;12: 79–87. doi:10.1017/S2040174420000045

9. English R, Peer N, Honikman S, *et al.* 'First 1000 days' health interventions in low- and middle-income countries: alignment of South African policies with high-quality evidence. Glob Health Action. 2017;10: 1340396. doi:10.1080/16549716.2017.1340396

10. South African Government. Child support grant. 2022, Available: https://www.gov.za/services/child-care-social-benefits/child-support-grant

11. South African Department of Health Department, Department of Performance Monitoring and Evaluation, Department of Social Development. Diagnostic/Implementation Evaluation of Nutrition Interventions for Children from Conception to Age 5: Final Report. Pretoria, South Africa: DoH, DDPME, DSD. 2014, Available: https://evaluations.dpme.gov.za/evaluations/441

12. Hasan MM, Magalhaes RJS, Ahmed S, *et al*. Meeting the Global Target in Reproductive, Maternal, Newborn, and Child Health Care Services in Low- and Middle-Income Countries. Global Health: Science and Practice. 2020;8: 654–665. doi:10.9745/GHSP-D-20-00097

13. Requejo J, Diaz T, Park L, *et al*. Assessing coverage of interventions for reproductive, maternal, newborn, child, and adolescent health and nutrition. BMJ. 2020;368: l6915. doi:10.1136/bmj.l6915

14. Bronfenbrenner U. Towards an experimental ecology of human development. American Psychologist. 1977;32: 513–531. doi:10.1037/0003-066X.32.7.513

15. Bronfenbrenner U. (1994) Ecological models of human development. International Encyclopedia of Education. Vol. 3, 2nd ed. Oxford: Elsevier.

16. Mahmudiono T, Segalita C, Rosenkranz RR. Socio-Ecological Model of Correlates of Double Burden of Malnutrition in Developing Countries: A Narrative Review. International Journal of Environmental Research and Public Health. 2019;16: 3730. doi:10.3390/ijerph16193730

17. Bhutta ZA, Das JK, Rizvi A, *et al.* Evidence-based interventions for improvement of maternal and child nutrition: what can be done and at what cost? Lancet. 2013;382: 452–477. doi:10.1016/S0140-6736(13)60996-4

18. Scott V, Schaay N, Schneider H, *et al.* Addressing social determinants of health in South Africa : the journey continues. South African Health Review. 2017;2017: 77–87.

19. Nussbaum MC, Sen A. The Quality of Life. Oxford: Oxford University Press; 1993.

20. Sen A. Inequality Reexamined. New York: Russell Sage Foundation; 1992.

For peer review only - http://bmjopen.bmj.com/site/about/guidelines.xhtml

21. Bronfenbrenner U. The ecology of human development: Experiments by nature and design. Cambridge, MA: Harvard University Press; 1979.

22. Ballard R, Hamann C. Income Inequality and Socio-economic Segregation in the City of Johannesburg. In: van Ham M, Tammaru T, Ubarevičienė R, Janssen H, editors. Urban Socio-Economic Segregation and Income Inequality: A Global Perspective. Cham: Springer International Publishing; 2021. pp. 91–109. doi:10.1007/978-3-030-64569-4_5

23. Statistics South Africa. Soweto. 2023, Available: https://www.statssa.gov.za/?page_id=4286

24. Hall K, Richter L, Mokomane Z, *et al.* South African Child Gauge 2018. Cape Town: Children's Institute, University of Cape Town; Available: http://www.ci.uct.ac.za/ci/child-gauge/2018

25. Cele L, Willen SS, Dhanuka M, *et al.* Ukuphumelela: Flourishing and the pursuit of a good life, and good health, in Soweto, South Africa. SSM - Mental Health. 2021;1: 100022. doi:10.1016/j.ssmmh.2021.100022

26. Ware LJ, Kim AW, Prioreschi A, *et al.* Social vulnerability, parity and food insecurity in urban South African young women: the healthy life trajectories initiative (HeLTI) study. J Public Health Pol. 2021;42: 373–389. doi:10.1057/s41271-021-00289-8

27. Department of Social Development. Children's Services Directory, Gauteng, 2016: A Guide to Provincial Non-Profit Organisations and Government Resources for Vulnerable Children. (2nd ed). Pretoria: USAID; 2016.

28. NGO Advisor Group. 2022, Available: www.ngoadvisor.co.za

29. Morse JM. The Significance of Saturation. Qual Health Res. 1995;5: 147–149. doi:10.1177/104973239500500201

30. MAXQDA 2020. Berlin, Germany: VERBI Software.; 2019. Available: https://www.maxqda.com/

31. Vaismoradi M, Turunen H, Bondas T. Content analysis and thematic analysis: Implications for conducting a qualitative descriptive study. Nursing & Health Sciences. 2013;15: 398–405. doi:10.1111/nhs.1204832.

32. Tong A, Sainsbury P, Craig J. Consolidated criteria for reporting qualitative research (COREQ): a 32-item checklist for interviews and focus groups. International Journal for Quality in Health Care. 2007;19: 349–357. doi:10.1093/intqhc/mzm042

33. National Interdepartmental Coordination Committee. National Integrated Food and Nutrition Security Plan 2018-2023. Pretoria, South Africa; 2017. Available: https://faolex.fao.org/docs/pdf/saf211944.pdf

34. National Department of Health. Strategic Plan for Maternal, Newborn, Child and Women's Health (MNCWH) and Nutrition in South Africa 2012-2016. Republic of South Africa: National Department of Health; 2012. Available: https://knowledgehub.health.gov.za/elibrary/strategic-plan-maternal-newborn-child-and-womens-health-mncwh-and-nutrition-south-africa#:~:text=in%20South%20Africa-,Strategic%20Plan%20for%20Maternal%2C%20Newborn%2C%20Child%20and%20Women's%20Health%20(,and%20the%20district%20health%20system.

For peer review only - http://bmjopen.bmj.com/site/about/guidelines.xhtml

35. Madhi SA, Thaele D, Sello M, *et al.* Patterns of healthcare utilisation and barriers affecting access to child healthcare services in low-income urban South African settings. South African Journal of Child Health. 2020;14: 34–39. doi:10.10520/EJC-20275dbeee

36. Solarin I, Black V. "They Told Me to Come Back": Women's Antenatal Care Booking Experience in Inner-City Johannesburg. Matern Child Health J. 2013;17: 359–367. doi:10.1007/s10995-012-1019-6

37. Lundberg O. Next steps in the development of the social determinants of health approach: the need for a new narrative. Scand J Public Health. 2020; 1403494819894789. doi:10.1177/1403494819894789

38. Dzau VJ, Laitner MH, Balatbat CA. Has traditional medicine had its day? The need to redefine academic medicine. The Lancet. 2022;400: 1481–1486. doi:10.1016/S0140-6736(22)01603-8

39. Dahab R, Sakellariou D. Barriers to Accessing Maternal Care in Low Income Countries in Africa: A Systematic Review. Int J Environ Res Public Health. 2020;17: 4292. doi:10.3390/ijerph17124292

40. Banke-Thomas OE, Banke-Thomas AO, Ameh CA. Factors influencing utilisation of maternal health services by adolescent mothers in Low-and middle-income countries: a systematic review. BMC Pregnancy and Childbirth. 2017;17: 65. doi:10.1186/s12884-017-1246-3

41. Erzse A, Goldstein S, Norris SA, *et al.* Double-duty solutions for optimising maternal and child nutrition in urban South Africa: a qualitative study. Public Health Nutr. 2020; 24:3674-3884. doi:10.1017/S1368980020002426

42. Besselink D, Brandt H, Klingberg S, *et al.* Perceptions of healthy food, and perceived facilitators and barriers to buying and consuming healthy food, among female caregivers in Soweto, South Africa. South African Journal of Child Health. 2022;16:172–177. doi:10.7196/SAJCH.2022.v16i3.1883

43. Drysdale RE, Slemming W, Makusha T, *et al.* Father involvement, maternal depression and child nutritional outcomes in Soweto, South Africa. Maternal & Child Nutrition. 2021;17: e13177. doi:10.1111/mcn.13177

44. National Planning Commission. National Development Plan 2030 Our Future-make it work. 2012, Available: https://www.gov.za/sites/default/files/gcis_document/201409/ndp-2030-our-future-make-it-workr.pdf

45. Maharaj P, Dunn S. "It's Not Easy Being a Mom Especially When You Are Unemployed": Navigating Childcare, a Necessary Social Service in South Africa. Journal of Social Service Research. 2022;48: 472–484. doi:10.1080/01488376.2022.2087826

46. Lee H-Y, Song IH, Kawachi I. Maternal and child social support and food availability in relation to child growth in four low- and middle-income countries. Sci Rep. 2022;12: 5910. doi:10.1038/s41598-022-09850-1

47. Mlotshwa L, Manderson L, Merten S. Personal support and expressions of care for pregnant women in Soweto, South Africa. Glob Health Action. 2017;10: 1363454. doi:10.1080/16549716.2017.1363454

For peer review only

48. Mannava P, Durrant K, Fisher J, *et al.* Attitudes and behaviours of maternal health care providers in interactions with clients: a systematic review. Globalization and Health. 2015;11: 36. doi:10.1186/s12992-015-0117-9

49. Silal SP, Penn-Kekana L, Harris B, *et al.* Exploring inequalities in access to and use of maternal health services in South Africa. BMC Health Services Research. 2012;12: 120. doi:10.1186/1472-6963-12-120

50. Tlebere P, Jackson D, Loveday M, *et al.* Community-Based Situation Analysis of Maternal and Neonatal Care in South Africa to Explore Factors that Impact Utilization of Maternal Health Services. Journal of Midwifery & Women's Health. 2007;52: 342–350. doi:10.1016/j.jmwh.2007.03.016

51. Sewpaul R, Crutzen R, Dukhi N, *et al.* A mixed reception: perceptions of pregnant adolescents' experiences with health care workers in Cape Town, South Africa. Reproductive Health. 2021;18: 167. doi:10.1186/s12978-021-01211-x

52. Lapham J, Martinson ML. The intersection of welfare stigma, state contexts and health among mothers receiving public assistance benefits. SSM Popul Health. 2022;18: 101117. doi:10.1016/j.ssmph.2022.101117

For peer review only

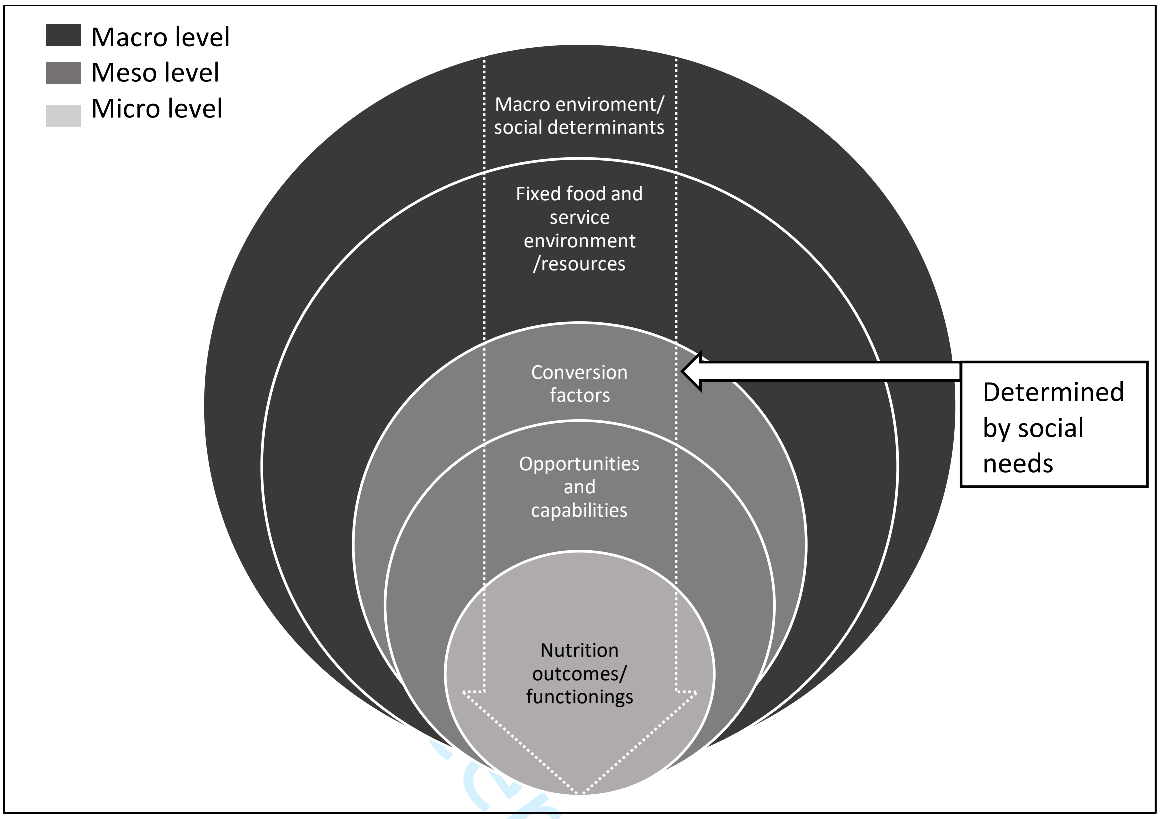

Figure 1. A social needs framework combining the capabilities approach (20) and the ecological model (22)

For peer review only

Supplemental file 1 - Total number of focus groups held by sociodemographic characteristics of participants

FGD no.	Participants (n)	Age (years) (min-max)	Pregnancy status	Participants with a household income <=3000 ZAR/month (n)	Participants with >=2 children (n)	Mean number of household members (SD)	Participants (n) per selected community health centres
1	7	22-40	Pregnant	5	1	4 (2.7)	Chiawelo (1), Itireleng (1), Lillian Ngoyi (-), Mofolo (3), Zola (2)
2	8	22-40	Not pregnant	4	7	5 (2.4)	Chiawelo (3), Itireleng (3), Lillian Ngoyi (-), Mofolo (2), Zola (2)
3	7	19-47	Not pregnant	6	5	4 (1.6)	Chiawelo (1), Itireleng (1), Lillian Ngoyi (1), Mofolo (3), Zola (1)
4	8	18-37	Pregnant	7	3	5 (2)	Chiawelo (1), Itireleng (3), Lillian Ngoyi (2), Mofolo (1), Zola (1)
5	8	26-49	Not pregnant	5	4	6 (2.7)	Chiawelo (1), Itireleng (2), Lillian Ngoyi (2), Mofolo (1), Zola (2)
6	7	22-48	Not pregnant	5	6	5 (2.8)	Chiawelo (1), Itireleng (3), Lillian Ngoyi (-), Mofolo (1), Zola (2)
7	6	19-42	Pregnant	3	2	4 (1.6)	Chiawelo (1), Itireleng (1), Lillian Ngoyi (2), Mofolo (2), Zola (-)

For peer review only

For peer review only - http://bmjopen.bmj.com/site/about/guidelines.xhtml

Supplemental file 2 - Semi-structured interview guide for employees from community-based organizations

Guidance for interviewer:

- Thank the participant's willingness to participate.
- Introduce yourself and the project, including i) the purpose for the interview, ii) who is involved in the process, iii) why the i participant's cooperation is important in collecting the information, iv) what will happen with the collected information and how the community will benefit.
- Explain that participation is completely voluntary, and the participant is free to decline/withdraw before or even after starting the interview process. If the interviewee says no, this will not have any negative consequences for the participant.
- Ensure that participant has provided written consent to participate and audio record the interview.

Interview questions:

- Could you please introduce yourself, tell me a little bit about who you are, where you are from? (*Probe: age, education background, how long have they been working at the organization, any children, where did they grow up, where do they live now.)*
- Can you describe your role at the organization?
- Could you please tell me about the projects / services that the organization runs? (*Probe: How do these interventions work? Think of impact pathways and mechanism).*
- How does your organization decide which activities it runs?
- How do you measure the success of programmes that are delivered in this community? *(Probe: what are some of the indicators used).*
- Can you tell me about the community that your organization works with?
- What are the greatest challenges facing pregnant women or women with young children in the community you are working with?
  (Probes:
  - *What do you think some of the reasons are that the challenge, such as: _________ happen?*
  - *Can you think of someone who is struggling with _________(e.g., unemployment)? What stopped her from finding employment? What have they done to overcome this?)*
- You talked about challenges, such as: _________. How does this challenge affect nutrition for pregnant women and infants and young children in this community?
- One of the challenges you talked about that affect nutrition was: _________. What are some of the needs mothers have to overcome this challenge? Do you know of services and resources that can help? *(Probe if the focus is on government/medical solution*: *Is there anything that could help in the community?)*
- I would like to hear your opinion about the following scenario: Let's say that two mothers receive the same service *(repeat a service mentioned)*. One mom can fully benefit and have good nutrition. The other mom is not able to turn the service into nutrition benefit. Can you think of reasons why this could be?
  (*Probes:*
  - *What limits the mom's ability to ____?*
  - *Is this a common issue in your community?*
  - *How can moms overcome this challenge?)*
- Do any of these services exist or are there others in place?

For peer review only - http://bmjopen.bmj.com/site/about/guidelines.xhtml

(If participants find it difficult to answer, provide examples and probe: in some settings, mothers who e.g. receive home-energy assistance programme or housing stability programme improve their nutrition. What would be relevant in this community?)

- How could these interventions work? What are the ways that it could make a difference? *(Probe: impact pathways and mechanism)*
- In my study I am using the term "social need" is this a term that you are familiar with? And the community? What is your understanding of this term? What other terms are used that might mean the same thing?
- Do you have any additional comments or information you would like to share that I have not asked you?

Guidance for interviewer: thank the participant their time and contribution. Provide reimbursement and ensure receipt of fund sheet has been signed.

Supplemental file 3 - Focus group discussion guide

Blue words provide the focus group discussion facilitator directions. These are not meant to be read aloud. Black words are meant to be read aloud.

INTRODUCTION

As we begin, let me tell you a bit about todays' discussion. We will focus on mom's, including pregnant women, and child nutrition. More specifically, we will talk about issues that **help** or **limit** moms' ability to keep themselves and their children well-nourished in your community.

This discussion is the second step in a bigger research project. In the first step we asked community-based organizations in Soweto about their experience of the different challenges and needs moms have when it comes to nutrition. This is because poor nutrition is common in Soweto. Some moms and children lack of important vitamins, some are undernourished some have obesity.

Community-based organizations helped us understand that the government tries to provide services to make sure that moms and children can get good nutritional care and support. There is help at the clinic level through supplementation and nutrition education, and there is also help from the government through the child support grants. However, we learnt that not every mom can benefit from these services the same way, and not every mom and child will be well nourished. This is because we are not the same. We face different challenges and have different opportunities. And these affect our ability to buy and eat nutritious food.

Today, we want to understand these challenges and find solutions with you to improve the nutrition for moms and children.

There will be 2 parts to our discussion.

First, you will be asked to talk about challenges that affect the nutrition of moms and pregnant women living in this community. Second, you will be asked to think of some of the resources or services that exist in your area that might help meet these needs and concerns.

For peer review only - http://bmjopen.bmj.com/site/about/guidelines.xhtml

There are no right or wrong answers. How does everyone feel about this so far? Do you have any questions? If not, please sign the consent form to participate and to audio record the discussion. Please write you name as well as your participant number on the forms.

Participants sing consent forms.

Before we begin, here are some ground rules. I am going to ask you keep your phone on silent/switch it off. I would also like to ask everyone, to speak as loud and clear as possible. Please, respect each other's opinions and give each other time to speak without disturbance. Only one person will be allowed to speak at a time. You can raise your hand if you have something to say and I will give you an opportunity. It is possible that you know someone who is here today. In this case, it is important that we all keep this discussion private.

Let participants know that you will start recording. Start recording. List place, date, time, group number and type (pregnant mothers or non-pregnant mothers).

PART 1

ICEBREAKER:
I'd like everyone to introduce themselves using your participant number and share how many kids you have, and what you heard about good nutrition from the clinic or anywhere else.

QUESTION 1:

As a next step, I would like to hear your opinion about the following scenario: Let's say that two mothers receive the same nutrition advice at the clinics. One mom is able to follow this advice and have good nutrition. The other mom is not able to do as she is told.

Can you think of reasons why this could be?

Probes: - What limits the mom's ability to follow the advice?
- *Is this a common issue in your community?*
- *How can moms overcome this challenge?*

QUESTION 2:

As we move on, I will read another scenario and ask you opinion.
It goes like: *"Your friend, Nomsa lives with her young child who has been losing weight. The supermarket to buy healthy food is an hour away by taxi. Nomsa is struggling to provide food for her hungry child. For months, they haven't eaten any fruits and vegetables, so they fill up with pap. This week she had to sell clothes to buy food."*

Is this something that happens in your community? Can you think of a solution? (if solution of "government" ask about local solutions).

Probes: - What do moms buy when they can't buy healthy food?
- *Can you think of someone who overcome this challenge?*

QUESTION 3:

We talked about X and Y that makes it difficult for a mother to have nutritious and healthy diet. What are other challenges can you think of that affect the nutrition of moms and pregnant women?

For peer review only - http://bmjopen.bmj.com/site/about/guidelines.xhtml

Probes:

- *"**unemployment**"→ How does not having a job affect your and your babies' nutrition?*

- *"**expensive** food"→ Are there other any other ways you could get nutritious food?*
  → What food do moms have if they can't get healthy food?

- *"**no healthy stores**"→ what makes it difficult to access supermarkets?*

- ***"little grant money"*** *→ You mentioned grants. Let me ask about a scenario when two poor mothers both receive the child support grant. For one of the mothers the grant lasts longer. For the other if finishes in a week. Can you think of the reasons why?*

How does *[e.g. difficulty to get to supermarkets; everyone selling vetkoeks; eating only pap and rice/vetkoek]* affect moms ability to follow the nutrition advice?

What else makes it difficult for a mother to **buy** nutritious and healthy diet?

What makes it difficult for a mother to **prepare** nutritious and healthy food?

What do others think about *[these needs/concerns]?*

PART 2 – LOCAL & COMMUNITY SOLUTIONS

Okay. We talked about a number of challenges, such as *(summarize what participants said)* let's focus more on local solutions. **We all know that the government could and should do more. But government solutions takes a long time.** While we are waiting for the government, **we need quick local solutions.**

We'll go around the table asking each person to pick a challenge we talked about and recommend an existing or imagined service to overcome the challenge.

Any questions before we start? *PAUSE for questions. Pick a person to start.*
Let's begin with you.

QUESTION 4:
You mentioned *(pick a challenge, e.g., difficult to go to the supermarket)* - what would be the solution to that?

Probes:

- ***"government should"*** → Besides the government solution, what can we do in the community?

- ***"more jobs"***→ Besides the government creating jobs, what can be done in the community? Do you know of mothers who were unemployed and now have jobs?

- ***"more grant money"***→ *You mentioned that often people don't know how to maintain the budget. Would more grant money be spent more wisely?*

- ***"More income"*** *→ can you think of community initiatives to overcome financial issues?*

For peer review only - http://bmjopen.bmj.com/site/about/guidelines.xhtml

- *"Less expensive food"* → *are there other ways to cut our spending on food in the shops?*

QUESTION 5: Follow up to mentioned solutions:

- **Do you know of any solution like that which exists in your community?**
- **Who should deliver this solution?**
- **What would it take to make this solution a reality?**
- **How does this solution help moms to ensure nutritious food?**

What do other people think? What else can be done?

QUESTION 6:

To what extent moms participate in community groups or community-based organization that can help address their challenge?

PART 3 - Closing the FGD

Once everyone has had a turn, ask the group if anyone wants to suggest other challenges, if they are satisfied with the solutions discussed.

- Have we covered the most important challenges around nutrition?
- How satisfied are you with the recommendations?
- Who would like to make any other recommendations?

Okay. I hope you are happy with the solutions and that you enjoyed this exercise. Thank you for your important insights. We hope to discuss your perspectives with your local stakeholders in the future. Thank you for your time. Before you leave, please go to my colleague, to collect travel reimbursement money and sign a registry.

Supplemental file 4 - Coding structure

Code	Description
**Theme: Available services and resources**	
Food aid	Food parcels and food vouchers distributed by the Department of Social Development, community-based organizations, churches, schools, and clinics.
Crèches	Childhood education centres that offer childcare services, including feeding schemes.
Stokvels	An invitation-only group of twelve or more people serving as a rotating credit union or saving scheme.
Skills training	Any learnership, fellowship programmes, volunteering opportunities in the community.
Clinic-based services	Services including nutrition advice, dietitian consultations, antenatal and postnatal care, supplementation, and social work support provided within clinical settings.
Poverty alleviation projects	Community projects such as knitting, sewing, and beading projects, baking groups and co-operatives in the community.
Food garden	Refers to food gardens located at schools, community-based organizations, and within households.

For peer review only - http://bmjopen.bmj.com/site/about/guidelines.xhtml

Social grants	Includes reference to the South African Child Support Grant, Disability Grant, Old Age Grant, Foster Child Grant, Care Dependency Grant, and Social Relief of Distress Grant.
**Theme: sources of variation**	
Delayed/periodic access to healthier food	Circumstances of delayed social grant payment, temporary access to healthier food upon income or social grant receipt. Includes references to running out of food budget by the end of the month.
Perceived and experienced mistrust, corruption, exclusion	Perceptions around unfairness in food aid distribution, local job recruitment including exclusion from food gardens due to young age and from economic opportunities due to old age.
Life skills	Refers to digital, technological, and internet literacy; challenges with CV writing, job search and application, and form completions.
Ability to pay for transportation, printing, internet	Transportation costs associated with access to supermarkets, government departments, skills training, and job opportunities.
School dropouts	Refers to dropping out of school often due to inadequate financial and childcare support for expectant and new mothers, and lack of necessary documentation for receipt of matric certificate.
Service eligibility	Possession of matric certificate, birth certificate, identification document.
Poor living conditions	Inadequate garden space, refrigeration, storage space and lack of stable electricity.
Household's food budget must cater for all vs individuals' need	Challenge of managing limited resources to provide food that satisfies everyone in the household while considering diverse nutritional needs of pregnant and lactating mothers, and young children.
Overcrowding, sharing of scarce resources	Refers to households with many members in limited space, leading to shorter durations of benefit from services like food parcels and vouchers, as resources must be divided among more people.
Competing household expenses	Refers to cases when healthier foods are sacrificed as budget is needed for monthly rent payment, school-related expenses (e.g., uniforms, stationary), maternity and baby clothing, toiletries, and pampers.
Lack of control over nutrition in the household	Refers to the inadequate control of mothers and pregnant women over household's nutrition budget and limited say in household food selection.
Grant-, kinship-, loan-dependency	Financial and livelihood dependency on income of household members, partners, elderly, loans, unfavourable relationships.
Single motherhood/absent fathers	Refers to cases where fathers are not actively engaged in providing material or emotional support to mothers and their children. Includes references to significant implications for the well-being and financial stability of mothers and children.
Poor partner and family dynamics	Represents a complex set of challenges related to familial and interpersonal relationships, including rejection, conflicts, and unsupportive behaviours.
Know-how of eating healthy on a budget	The knowledge and skills required to maintain a nutritious diet while managing limited financial resources. It includes strategies such as using savings to incorporate variety into one's diet, preparing meals at home to save money, seeking discounts and deals when shopping, and making cost-effective purchases by buying items in bulk.

For peer review only

Awareness of healthier diet	Refers to a combination of factors, including familiarity with a varied and balanced diet, understanding of nutrition beyond fruits and vegetables, willingness, and desire to incorporate variety into one's dietary.
Ability to trade off present investment for future return	Refers to financial investment (e.g., stokvels) and time investment (e.g., skills training).
Healthcare and welfare stigma	Encompasses the subjective sense of experiencing negative attitudes or societal judgment in situations involving healthcare interactions, teenage pregnancy, or seeking help from the community.
Ability to save, maintain and multiply budget	Refers to a range of skills and strategies, including budgeting expertise, income generation through garden production and entrepreneurship, and participation in community savings groups like stokvels. It reflects the capability to effectively manage and grow financial resources through various means.
Poor coping (mental health and substance abuse)	reflects the struggle to effectively manage stress and life difficulties, resulting in negative mental health outcomes and engagement in harmful behaviours like debt accumulation, gambling, and substance abuse.
Low self-esteem and hopelessness	Feelings of being stuck, sense of hopelessness, and a lack of motivation, seeing a "way out". In the context of motherhood, it refers to a lack of knowledge about pregnancy and parenting, leading to negative influences on how mothers perceive their own capabilities and effectiveness in their parenting role.
Domestic violence	This includes child neglect, gender-based violence, financial, emotional abuse within the household.
Influence of local food environment	Rerefers to choosing unhealthy food over healthy due to cost, the abundance of convenient but unhealthy produce, and the lack of locally available fresh and healthy alternatives. Includes reference to long distance from supermarkets, compromising quality and quantity of food purchased.
Social connectedness	Reflects the level of involvement and connection of mothers and pregnant women within their social environment. Includes engagement in self-help groups, awareness of community resources and services, participation in savings clubs (stokvels), and church groups.
Lack of belonging, isolation	Refers to the experience of feeling disconnected, alone, or unsupported (financially and emotionally) in the role of motherhood. It reflects the absence of a supportive peer network and emotional closeness, contributing to a sense of isolation and detachment.
**Theme: Social needs**	
Affordable healthier foods	Links to theme 2 codes: Delayed/periodic access to healthier food, Ability to pay for transport to supermarkets, Awareness of healthier diet (beyond fruits and vegetables i.e., legumes, brown rice), Influence of local food environment.
Social support	Includes psychosocial support that links to theme 2 codes: Healthcare and welfare stigma, Low self-esteem and hopelessness, Poor coping (mental health and substance abuse), Poor partner and family dynamics, Domestic violence.<br>Further, it includes social cohesion and connectedness that links to theme 2 codes: Perceived and experienced mistrust, corruption, exclusion, Lack of belonging, isolation, Social connectedness (i.e., group membership).
Access to civic services, welfare, and labour market	Service eligibility (i.e., possession of identification documents, birth certificate, matric certificate); School dropouts; Life skills (i.e., internet

For peer review only

	literacy, CV writing, form completion); Ability to pay for transportation, printing, internet
Appropriate and affordable housing	Links to theme 2 codes: Monthly rent payments; Overcrowding, sharing of scarce resources; Household's food budget must cater for all vs individuals' nutritional needs; Poor living conditions (i.e., lack of stable electricity, storage space, refrigeration)
Personal income stability	Links to theme 2 codes: single motherhood/absent fathers, Grant-, kinship-, loan-dependency, Lack of control over nutrition in the household.
Financial planning for nutrition	Links to theme 2 codes: Ability to save, maintain and multiply budget; Ability to trade off present investment for future return; Know-how of eating healthy on a budget (i.e., buying in bulk, home preparation)

Supplemental file 5 - Consolidated criteria for reporting qualitative studies (COREQ): 32-item checklist

Developed from: Tong A, Sainsbury P, Craig J. (2007) Consolidated criteria for reporting qualitative research (COREQ): a 32- item checklist for interviews and focus groups. International Journal for Quality in Healthcare: 19:349 – 357

No. Item	Guide questions/description	Reported on Page #
**Domain 1: Research team and reflexivity**		
Personal Characteristics		
1. Interviewer/facilitator	Which author/s conducted the interview or focus group?<br><br>*Interviews were conducted by AE, and SN (see in acknowledgements) facilitated the focus groups.*	7
2. Credentials	What were the researcher's credentials? E.g. PhD, MD<br><br>*AE – MSc*<br>*SN - Matric exemption certificate*	N/A
3. Occupation	What was their occupation at the time of the study?<br><br>*AE – Senior Researcher*<br>*SN – Qualitative research assistant*	N/A
4. Gender	Was the researcher male or female?<br><br>*Both interviewers identified as women.*	N/A
5. Experience and training	What experience or training did the researcher have?<br><br>*AE and SN had experience in qualitative interviewing and working with members of the community in the study setting.*	7
Relationship with participants		
6. Relationship established	Was a relationship established prior to study commencement?<br><br>*The AE and SN were unknown to participants prior to the commencement of the study and recruitment.*	N/A

For peer review only - http://bmjopen.bmj.com/site/about/guidelines.xhtml

7. Participant knowledge of the interviewer	What did the participants know about the researcher? e.g. personal goals, reasons for doing the research<br><br>*AE and SN introduced themselves at beginning of the interviews and focus groups, explained their roles, occupations, and purpose of the research.*	Supplemental file 2 and 3
8. Interviewer characteristics	What characteristics were reported about the inter viewer/facilitator? e.g. Bias, assumptions, reasons and interests in the research topic<br><br>*See above*.	Supplemental file 2 and 3
**Domain 2: Study design**		
Theoretical framework		
9. Methodological orientation and Theory	What methodological orientation was stated to underpin the study? e.g. grounded theory, discourse analysis, ethnography, phenomenology, content analysis<br><br>*A novel theoretical framework was developed for the study that informed data collection and analysis. Thematic analysis was used to analyse the data.*	4-6
Participant selection		
10. Sampling	How were participants selected? e.g. purposive, convenience, consecutive, snowball<br><br>*Purposive sampling.*	6
11. Method of approach	How were participants approached? e.g. face-to-face, telephone, mail, email<br><br>*To approach interview participants, an invitation letter was sent to their respective community-based organization, followed up with a telephone call. Focus group discussion participants were approach in person at health care facilities.*	6
12. Sample size	How many participants were in the study?<br><br>*18 employees representing 10 community-based organization were interviewed, and 51 pregnant women and mothers participated in focus groups.*	8
13. Non-participation	How many people refused to participate or dropped out? Reasons?<br><br>*None*.	N/A
Setting		
14. Setting of data collection	Where was the data collected? E.g. home, clinic, workplace<br><br>*The interviews were conducted either in person at the community-based organization or via telephone. Focus groups took place at a research centre in the community.*	7
15. Presence of non-participants	Was anyone else present besides the participants and researchers?	N/A

For peer review only - http://bmjopen.bmj.com/site/about/guidelines.xhtml

	No.	
16. Description of sample	What are the important characteristics of the sample? e.g. demographic data, date<br><br>*Please refer to Table 2.*	8 and Table 2
Data collection		
17. Interview guide	Were questions, prompts, guides provided by the authors? Was it pilot tested?<br><br>*Yes, please refer to supplemental file 2 and 3.*	6 and supplemental file 2 and 3
18. Repeat interviews	Were repeat interviews carried out? If yes, how many?<br><br>*No.*	N/A
19. Audio/visual recording	Did the research use audio or visual recording to collect the data?<br><br>*Yes, the interviews and focus groups were audio-recorded with consent from participants.*	6
20. Field notes	Were field notes made during and/or after the interview or focus group?<br><br>*Memos in the form of digital notes were taken during the data analysis process to help with theme generation and refinement.*	N/A
21. Duration	What was the duration of the inter views or focus group?<br>*The interviews and focus groups lasted about an hour.*	7
22. Data saturation	Was data saturation discussed?<br><br>*Transcripts were analysed progressively to determine the point at which saturation was achieved.*	7
23. Transcripts returned	Were transcripts returned to participants for comment and/or correction?<br><br>*No.*	N/A
**Domain 3: analysis and findings**		
Data analysis		
24. Number of data coders	How many data coders coded the data?<br><br>*One (AE).*	7
25. Description of the coding tree	Did authors provide a description of the coding tree?<br><br>*Yes, please refer to supplemental file 4.*	7 and supplemental file 4
26. Derivation of themes	Were themes identified in advance or derived from the data?<br><br>*Themes were identified through a hybrid (deductive and inductive) approach.*	7
27. Software	What software, if applicable, was used to manage the data?	7

For peer review only - http://bmjopen.bmj.com/site/about/guidelines.xhtml

	MAXQDA 2022 data analysis software.	
28. Participant checking	Did participants provide feedback on the findings?<br><br>*A summary of the findings was presented to members of the same community in the next phase of the bigger research projects. Community members (different from the present study's participants) further engaged with the findings and informed the larger study's objectives.*	N/A
Reporting		
29. Quotations presented	Were participant quotations presented to illustrate the themes/findings? Was each quotation identified? e.g. participant number<br><br>*Yes, interview participant quotations were identified by gender, role, and organization number. Focus group participants were identified by pregnancy status and focus group number. All findings were illustrated with relevant quotations.*	11-14
30. Data and findings consistent	Was there consistency between the data presented and the findings?<br><br>*Yes, all data was interpreted in the Discussion section and was linked to existing literature.*	14-16
31. Clarity of major themes	Were major themes clearly presented in the findings?<br><br>*Yes.*	9-13
32. Clarity of minor themes	Is there a description of diverse cases or discussion of minor themes?<br><br>*Minor themes were discussed as part of each major theme. We identified only minor instances of differing perspectives amongst participants. These were reported as part of the relevant themes. Please refer to page 13 for an example.*	13

For peer review only
